# Supplementary material for: The effectiveness of high-intensity interval training versus moderate intensity continuous training in prehabilitation among patients undergoing major abdominal surgery: A study protocol
Source: PLoS One. 2025 Oct 10;20(10):e0332361. doi: 10.1371/journal.pone.0332361 (PMC12513630; doi:10.1371/journal.pone.0332361)
Supplement: S2 File — (PDF) [file pone.0332361.s002.pdf]

The Effectiveness of High-Intensity Interval Training versus Moderate Intensity Continuous Training in Prehabilitation among Patients Undergoing Major Abdominal Surgery.

MASTER STUDENT:

SURIAH BINTI AHMAD (P134844)

Main SUPERVISOR:

Dr. Nor Azura Binti Azmi Pensyarah Universiti Pusat Kajian Rehabilitasi & Keperluan Khas (iCaReHab) nazura.azmi@ukm.edu.my

Name and Institution of the principal investigator

Prof Madya Dato' Dr Nur Ayub Mohd Ali

Cardiothoracic Surgeon

Department of Surgery

Hospital Canselor Tuanku Muhriz

Dr. Nor Azura Binti Azmi

Pensyarah Universiti

Pusat Kajian Rehabilitasi & Keperluan Khas (iCaReHab)

Fakulti Sains Kesihatan

Universiti Kebangsaan Malaysia

Name and Institutions of Co-Investigators

Dr. Katijjahbe Binti Mohd. Ali

Pegawai Pemulihan Perubatan Jab

Perkhidmatan Pemulihan Perubatan,

Hospital Canselor Tuanku Muhriz

Name and Institutions of Co-Investigators

Dr. Chik Ian

Hepatobiliary Surgeon

Department of Surgery

Hospital Canselor Tuanku Muhriz

Dr Sharifah Noor Nazihah binti Sayed Masri

Anaesthesiologist

Department of Anaethesiology

Hospital Canselor Tuanku Muhriz

Name and Address of sponsor

Self-sponsored

Study site

Hospital Canselor Tuanku Muhriz Cheras
(HCTM), Kuala Lumpur

Table of Content

1.0 Introduction

1.1 Research Background

1.2 Research Question

1.3 Objectives

1.4 Hypothesis

1.5 Expected Outcome and Benefit

2.0 Research Methodology

2.1 Study Type Design

2.2 Study Period

2.3 Study Location

2.4 Study Population

2.5 Sampling and Sample Size Calculation

2.6 Data Collection

2.8 Statistical Analysis Plan

2.9 Flow Chart

3.0 Ethical Issue

3.1 Ethics of study

3.2 Informed consent/assessment process

3.3 Privacy and confidentiality

3.4 Conflict of interest

3.5 Publication policy

3.6 Termination of study

4.0 Gantt Chart and Milestone

5.0 Budget

6.0 References

7.0 Appendices

Abstract

Background

Prehabilitation often referred to as a fit-for-surgery program in the form of body conditioning exercises aims to optimize the patient's condition before surgery. However, the precise protocol of prehabilitation has not been completely established.

Objective

This study aims to (i) develop prehabilitation of intensity training protocol and (ii) evaluate the potential effects of high-intensity interval training (HIIT) compared to moderate intensity continuous training (MICT) among patients undergoing major abdominal surgery.

Methods

A two-phase research design will be executed: (i) the first phase is a cross-sectional web survey will be conducted to explore the current practice amongst physiotherapists treating patients following major abdominal surgery in Malaysia hospitals and eventually develop a protocol for prehabilitation program; and (ii) the second phase will be a prospective, double-blinded randomized controlled trial (RCT) which aims to determine the potential effect of HIIT on multi-domain recovery. For the web survey, 74 Malaysian physiotherapists will be recruited to complete this survey. For the RCT, a total of 70 participants will be recruited from the surgery and anaesthetic clinic at the Hospital Canselor Tunku Muhriz. Participants will be randomly allocated to either receive HIIT or MICT (control group) with 35 participants in each group. Both groups will receive body conditioning and respiratory muscle strength exercises. For the intervention group, participants will receive HIIT and the control group MICT. This will be one-hour therapist-supervised intervention sessions for at least 4 weeks of exercise duration with 2 sessions per week before the surgery. The patient will be assessed at baseline, before the operation, before discharge and 4 weeks postoperatively and 3 months postoperatively. The outcomes will be assessed using multi-domain recovery measures that include physical measures (6MWT), Short Physical Performance Battery and 1 minute Sit to Stand), upper limb strength (Hand Grip Test), fatigue (Fatigue Severity Scale), psychological recovery (the Hospital Anxiety Depression Scale (HADS) and health-related quality of life (EuroQoL). Data will be analysed using intention-to-

treat analysis. The changes from all timelines in the secondary outcomes will be analysed using the Mixed Model ANOVA test.

Result: The findings of this research will inform guidelines of prehabilitation in determining the optimal dosage of exercise prescriptions/intensity for patients undergoing major abdominal surgery.

Conclusion:
The protocol of this study is registered in the Australia New Zealand Clinical Trial Registry with registration number ACTRN12625000023459.

Keywords : Major abdominal surgery, prehabilitation, body conditioning exercise, high intensity interval training, moderate intensity exercise training

Introduction

Abdominal surgery is the most common surgery performed worldwide [1]. Elective major abdominal surgery (MAS) is defined as a planned of any abdominal surgery where the total incision length is ≥ 5 cm involving open, laparoscopic, or minimally invasive procedures performed under general anaesthesia, with an incision into the abdominal cavity, visceral manipulation, and at least an overnight hospital stay [1].

The most common complication following MAS is a postoperative pulmonary complication (PPCs) with a reported incidence of 13–53 % [2]. Most PPCs manifest within the initial 3 days following surgery [3,4]. Evidence indicates that PPCs are associated with prolonged hospital stays [5,6], an elevated risk of unplanned readmissions [5], diminished quality of life and physical function [7], and an increased risk of all-cause mortality within 12 months [2,5]. Owing to the high incidence of PPCs and their substantial impact on patients, the prediction and targeted prevention of PPCs are strongly recommended [8].

Enhanced Recovery after Surgery (ERAS) is an evidence-based preoperative, intraoperative and postoperative multi-model care pathway that aims to reduce stress response to surgery and accelerate post-op recovery through enhancing patient mobilization, reducing complication rates after surgery, decreasing hospital length of stay and reducing healthcare costs [1,9,10]. Since ERAS was first implemented within hospitals over twenty years ago, post-surgical outcomes have improved for patients [11]. Length of stay has decreased, with no subsequent increase in readmission rates [12], with concurrent improvements in clinical outcomes whilst having a beneficial impact on healthcare resources. ERAS originated in elective colorectal surgery but has spread to other surgical subspecialties, including, but not limited to, gastrointestinal, hepatobiliary, orthopaedic, cardiac, thoracic, head and neck, breast and gynaecologic surgery [13]. The ERAS approach comprises preoperative, intraoperative, and postoperative components, with optimization of the patient's physical status before surgery as a key focus [9]. Therefore, prehabilitation program by physiotherapists is essential in enhancing patients' recovery and improving their outcomes after surgery including physical performances, psychological and health related quality of life [2,14,15].

The role of physiotherapy within ERAS pathways is important in both preoperative and postoperative routines [16]. Implementing a preoperative strength programme has been shown to

promote musculoskeletal improvements in preparation for a forthcoming physiological stressor, and is an emerging key component of ERAS [16]. A literature review found preoperative exercise in patients scheduled for cardiovascular, thoracic, abdominal and major joint replacement surgery to be well-tolerated and effective [17]. Postoperative exercise programmes are also recommended by ERAS guidelines, promoting muscle hypertrophy and the return to function after major surgery [18].

The standard prehabilitation program may include aerobic exercise which often moderate-intensity continuous training (MICT) is used to improve cardiovascular fitness [29], resistance training to strengthen muscles and improve overall physical function [16], breathing exercise to enhance lung capacity and respiratory muscle strength [20], and education with lifestyle counselling includes guidance on smoking cessation, nutrition, and overall physical activity [21]. High-intensity interval training (HIIT) is a bolus-dosing approach that efficiently increases cardiorespiratory fitness (CRF) and is feasible in most surgical populations. High-intensity interval training involves repeated aerobic high-intensity intervals at approximately 80% of the maximum heart rate, followed by active recovery [22,23,24]. The rapid increases in CRF elicited with HIIT is appealing for preoperative patients, and in the context of pathology, age, and comorbidities, the volume of training stimulus required to improve CRF can often be achieved [24].

HIIT provides advantages in terms of time efficiency, greater cardiorespiratory fitness, improved metabolic health and more effectiveness in preserving muscle mass. Studies demonstrated that HIIT, required significantly less time than MICT, produced similar cardiovascular and metabolic health improvements [25,26,27]. The study highlighted the time efficiency of HIIT as a major advantage for patients with limited time before surgery. The study by Weston et al. (2014) showed that HIIT led to greater improvements in VO_2 max compared to MICT in patients with cardiovascular disease [28]. Given that VO_2 max is a key indicator of cardiorespiratory fitness, HIIT's superior outcomes highlight its potential benefit in prehabilitation [28].

The current literature highlights the significant impact of prehabilitation on improving patient outcomes in the context of major abdominal surgery. Prehabilitation programs incorporating aerobic, resistance, and breathing exercises, alongside education and lifestyle counselling, have been shown to enhance cardiorespiratory fitness, reduce postoperative complications, and expedite recovery. While moderate-intensity continuous training (MICT) is

commonly used in such programs, high-intensity interval training (HIIT) has emerged as a time-efficient and potentially superior alternative, particularly in improving VO_2 max and preserving muscle mass.

Despite these findings, there is a lack of standardized exercise protocols tailored to the prehabilitation phase for patients undergoing major abdominal surgery. Additionally, limited data exist on the health status and functional outcomes of these patients throughout the surgical timeline. This gap warrants further investigation into the comparative effectiveness of HIIT and MICT, particularly in terms of their impact on multidomain recovery. By addressing these research gaps, this study protocol aims to provide valuable insights into optimizing prehabilitation strategies, ultimately improving the quality of care and recovery outcomes for patient

s undergoing major abdominal surgery.

Therefore, this study intended to evaluate the effectiveness of HIIT over MICT prehabilitation training on cardiorespiratory fitness and maximum inspiratory pressure among patients undergoing major abdominal surgery. This study also aims to investigate the effectiveness of HIIT versus MICT prehabilitation training on physical performances, upper limb strength, body composition, fatigue, psychological and health-related quality of life. We hypothesize that a 4-week HIIT program during prehabilitation is non-inferior to MICT in improving the targeted outcomes following major abdominal surgery, meaning the difference between the two interventions does not exceed the pre-specified non-inferiority margin (Δ).

Literature Review

Abdominal surgery is the most common surgery performed worldwide. Major abdominal surgery is conducted under general anaesthesia and involves making an incision into the abdominal cavity with manipulation of the internal organs, typically requiring at least an overnight hospital stay (Donati et al., 2004). Physiotherapists studying this population often classify abdominal surgeries based on the size and location of the abdominal incision(s) (Reeve, 2016). Under this definition, major abdominal surgery encompasses gastrointestinal, hepatobiliary, colorectal, urological, vascular, and

gynaecological procedures performed via open or minimally invasive abdominal approaches, but generally excludes laparoscopic surgeries where the total incision length is less than 5 cm

Mounting evidence has firmly established that low levels of cardiorespiratory fitness (CRF) are associated with a high risk of cardiovascular disease, all-cause mortality, and mortality rates attributable to various cancers. Recent studies suggest that in addition to being a strong predictor of cardiovascular and all-cause mortality in both asymptomatic and clinically referred populations, CRF could be especially helpful in the preoperative risk assessment of patients undergoing cardiovascular and noncardiovascular surgery, predicting surgical complications and short-term outcomes in patients subjected to abdominal aortic aneurysm repair, hepatic transplantation, lung cancer resection, upper gastrointestinal surgery, intra-abdominal surgery, bariatric surgery, and coronary artery bypass grafting. In addition, when patients with coronary artery disease who had to wait in the hospital for coronary artery bypass grafting were randomized into an exercise training group, outcomes were superior to those in the standard care group, because of a reduced rate of postoperative complications and shorter hospital stays were observed (Ross 2016)

Early Recovery after Surgery (ERAS) is a multi-model care pathway that reduces stress response to surgery and tries to accelerate post-op recovery. ERAS is managing the patient preoperatively, through the Operation and post-operatively. Enhanced recovery after surgery (ERAS) is a combination of perioperative care components built upon a multimodal approach that integrates evidence-based interventions to reduce convalescences across multiple surgical procedures. Since ERAS was first implemented within hospitals over twenty years ago, post-surgical outcomes have improved for patients (Kehlet and Wilmore 2008). Length of stay has decreased, with no subsequent increase in readmission rates (Paton et al. 2014), with concurrent improvements in clinical outcomes whilst having a beneficial impact on healthcare resources. ERAS programmes are supported by evidence-based preoperative, intraoperative and postoperative procedures to accelerate the achievement of discharge criteria. ERAS originated in elective colorectal surgery but has spread to other surgical subspecialties, including, but not limited to, gastrointestinal, hepatobiliary, orthopaedic, cardiac, thoracic, head and neck, breast and gynecologic surgery (Koci'an P 2023)

The role of physiotherapy within ERAS pathways is important in both preoperative and postoperative routines (Carli et al. 2010). Implementing a preoperative strength programme has been shown to promote musculoskeletal improvements in preparation for a forthcoming physiological stressor, and is an emerging key component of ERAS (Carli et al. 2010). A literature review found preoperative exercise in patients scheduled for cardiovascular, thoracic, abdominal and major joint

replacement surgery to be well-tolerated and effective (Hoogeboom et al. 2014). Postoperative exercise programmes are also recommended by ERAS guidelines, promoting muscle hypertrophy and the return to function after major surgery (ERAS Society 2017).

The standard prehabilitation program may include aerobic exercise which often moderate-intensity continuous training (MICT) is used to improve cardiovascular fitness (Moran et al 2016), resistance training to strengthen muscles and improve overall physical function (Carli et al 2010), breathing exercise to enhance lung capacity and respiratory muscle strength (Weiner et al 2002), and education with lifestyle counseling includes guidance on smoking cessation, nutrition, and overall physical activity (Barberan-Garcia et al. 2014)

A research done by Steffen et al. (2023), the preoperative exercise and education program will be delivered 4-8 weeks before surgery and include up to 24 sessions with 50 minutes per session with supervised, high-intensity training. Individualised exercise prescription, progression, and follow-up with a local physiotherapist or exercise physiologist up to 32 sessions for 30 minutes while unsupervised home exercise, up to 56 walking sessions for 30 minutes had been delivered plus advice to walk continuously for 30 minutes daily. Pablo et al (2023) concluded that prehabilitation programmes with more than one supervised session per week improved physical function but did not enhance surgical outcomes.

Inspiratory muscle training using an inspiratory threshold loading device for 2–4 weeks and indicated before abdominal or cardiac surgery to prevent postoperative pulmonary complications. Clinically significant complications include atelectasis, infection (bronchitis, pneumonia), prolonged mechanical ventilation and respiratory failure, exacerbation of underlying chronic lung disease, and bronchospasm (Katsura 2015). Preoperative inspiratory muscle training reduces the incidence of postoperative pneumonia and atelectasis (risk ratio 0.53; 95% CI 0.34–0.82). It also reduces the length of hospital stay by about one day (MD –1.33; 95% CI –2.53 to –0.13)(Katsura 2015).

A meta-analysis of prehabilitation interventions consisting of inspiratory muscle training, aerobic exercise, and/or resistance training found that prehabilitation decreased post-operative complications after intra-abdominal operations in a traditional surgical care setting (OR 0.59, 95% CI 0.38 to 0.91; p=0.03) (Moran 2016).

Chan (2021) proved that preoperative breathing exercises reduced length of stay (LOS), postoperative pulmonary complication (PPC), and pneumonia and potentially improved six minute walking distance (6MWD) in patients undergoing surgical lung cancer resection. Breathing exercises in combination with aerobic exercise yielded greater reductions in LOS. Randomized controlled trials are needed to test the feasibility of introducing a preoperative breathing exercise program in this patient population.

Exercise training is the intervention in this review. Exercise training is "a subset of physical activity that is planned, structured, and repetitive, and has as a final or an intermediate objective, the improvement or maintenance of physical fitness" (Caspersen 1985). This includes aerobic training, resistance training or a combination of these with or without inspiratory muscle training. Exercise training was not commonly prescribed in the preoperative management of people with NSCLC (Cavalheri 2013), possibly due to uncertain evidence of its feasibility and eHectiveness (Cavalheri 2020). However, the evidence for the eHectiveness of preoperative exercise training in people with NSCLC has substantially grown since the early 2010s. Cochrane Review in 2017, demonstrated initial evidence (low quality) that preoperative exercise training may reduce the risk of postoperative pulmonary complications, intercostal catheter duration and length of hospital stay, and may improve preoperative exercise capacity and forced vital capacity (FVC) (Cavalheri 2017).

Preoperative exercise can reduce postoperative hospital stay, pulmonary, and other complications in abdominal and cardiovascular surgery. PaO2 was significantly increased in the intervention group during the preoperative period following training. Exercise improves inspiratory muscle endurance, functional mobility, reduces postoperative pain scores and anxiety, and improves quality of life (Iqbal 2019). Matassi 2018 indicated that preoperative exercise of the arthritic knee facilitates immediate postoperative recovery following primary total knee replacement. Preoperative exercise therapy might also improve the physical fitness of patients before major surgery, and preoperative chest physiotherapy seems to be effective in reducing pulmonary complications (Pouwels 2014).

Prehabilitation enables patients to withstand the stress of surgery by augmenting functional capacity. Preoperative exercise decreases sympathetic over-reactivity, improves insulin sensitivity, and increases the ratio of lean body mass to body fat. It also improves physical and psychological readiness for surgery, reduces postoperative complications and the length of stay, and improves the

transition from the hospital to the community. A cardiac prehabilitation program should include education, nutritional optimization, exercise training, social support, and anxiety reduction, although current existing evidence is limited. Three non-CS studies have successfully demonstrated the benefits of 3 to 4 weeks of prehabilitation in the context of ERAS. Prehabilitation interventions prior to CS must be further examined to advance this area of research.

Prehabilitation before abdominal surgery has increased in popularity over recent years and aims to improve pre-operative conditioning of patients to improve post-operative outcomes. this is recommended as part of ERAS guidelines. The beneficial effect of such protocols is not well established with conflicting results reported. This review aimed to assess the effect of prehabilitation on postoperative outcomes after major abdominal surgery.

Moderate intensity interval training (MICT) is often chosen as the control in studies comparing different exercise modalities because it is a well-established, evidence-based component of prehabilitation programs. It involves sustained exercise at a moderate intensity, typically around 40-60% of VO2 max, which is safe and effective for improving cardiovascular and respiratory function. MICT is the traditional standard against which newer or alternative interventions, like High-Intensity Interval Training (HIIT), are compared.

MICT is normally has been chosen as a control because MICT has a long history of safe and effective use in various patient populations, including those awaiting surgery. West et al. (2015) demonstrated in a randomized controlled trial that MICT improved preoperative fitness levels and reduced postoperative complications in high-risk surgical patients. The study supported the use of MICT as a safe and effective intervention in prehabilitation. Besides that, it provides a baseline against which the benefits of more intense or novel interventions can be measured. In a systematic review, Hulzebos et al. (2012) used MICT as the control intervention to compare the effects of different preoperative exercise regimens. The study found that MICT consistently provided a reliable baseline for comparing the benefits of more intensive or alternative exercise programs. Furthermore, patients are generally more familiar with moderate-intensity exercise, which may lead to higher compliance rates in a clinical setting. A study by Lee et al. (2018) found that patients were more likely to adhere to MICT regimens due to their familiarity with moderate-intensity exercise. The

study emphasized that patient compliance is higher when the exercise intensity is perceived as manageable, making MICT a suitable control in prehabilitation studies.

High-intensity interval training (HIIT) is a bolus-dosing approach that efficiently increases Cardiorespiratory fitness and is feasible in most surgical populations. High-intensity interval training involves repeated aerobic high-intensity intervals at approximately 80% of the maximum heart rate, followed by active recovery. The rapid increases in CRF elicited with HIIT is appealing for preoperative patients, and in the context of pathology, age, and comorbidities, the volume of training stimulus required to improve CRF can often be achieved.

HIIT provide advantages more in term of time efficiency, greater improvement in cardiorespiratory fitness, improves metabolic health and more effective in preserving muscle mass. A study by Gillen et al. (2016) demonstrated that HIIT, which required significantly less time than MICT, produced similar improvements in cardiovascular and metabolic health. The study highlighted the time-efficiency of HIIT as a major advantage for patients with limited time before surgery.

The study by Weston et al. (2014) showed that HIIT led to greater improvements in VO2 max compared to MICT in patients with cardiovascular disease. Since VO2 max is a critical measure of cardiorespiratory fitness, HIIT's superior results suggest its potential advantage in prehabilitation.

Besides that, A meta-analysis by Jelleyman et al. (2015) concluded that HIIT was more effective than MICT in improving insulin sensitivity and reducing abdominal fat. These findings are particularly relevant for patients with metabolic conditions awaiting surgery. While, a study by Robinson et al. (2017) found that HIIT was more effective in preserving muscle mass in older adults compared to MICT. Muscle preservation is crucial for patients undergoing major surgery, as it can impact postoperative recovery and physical function.

In a systematic review and meta-analysis by Clifford (2023), pooled results indicated several positive associations of HIIT vs standard care with CRF or postsurgical outcomes. These findings suggest that HIIT may improve patient outcomes, with robust benefits across patient populations (Clifford et al. 2023) . Preoperative HIIT shows promising results and should be included in prehabilitation programs. The high degree of heterogeneity in our analysis demonstrates differences

in training programs and supports the need for further well-designed studies to improve the quality of evidence

Author	Population	Findings	Intervention	Outcome measure	Gap
Devin 2018 (RCT)	Colorectal ca survivor (n=57)	HIIE promotes superior improvements and short-term maintenance of V˙O2peak and fat mass improvements. HIIE training at a reduced frequency also promotes maintainable cardiorespiratory fitness improvements.	-MICE-50 minutes of cycling at 50-70% peak heart rate, 3x/week -HIIE-10 minute warm up at 50-70% Hrpeak, 4 minute interval, cycling at 85-95% Hrpeak,3 minute period of active recovery, repeated 4x, total of 38 min/session	VO2 peak<br>Fat mass	The research was based on pre operatively. No pre and post operative data regarding patients physical performance and quality of life
Bhatia C, 2019 (RCT)	Lung ca<br>(n=151) non-small cell lung cancer	Short-term HIIT was feasible and safe in preoperative setting and increased cardiorespiratory fitness	-HIIT-of 2–3-weekly, 2 × 10-min series of cycling at peak power, measured with CPET prior to training,15s on-off duty cycle, 5-min warm-up, 5min cool-down	Cardiorespiratory fitness (VO2peak), 6MWT, oxygen saturation, leg fatigue, and dyspnea (BORG scale	This research did not mention about the usual care they practice in the control group.
Egegaard T, 2019 (RCT)	(n=15)	High intensity was feasible, safe, and well tolerated during concomitant chemoradiotherapy; no significant differences within or between groups in any secondary outcome	moderate-to-high intensity aerobic interval Exercise training -20min training 5x/week	(VO2peak), functional capacity (6MWD), pulmonary function (FEV1), psychosocial parameters (quality of life (FACT-L), anxiety and depression (HADS)	- Small sample size
Sara Mijwell 2018 (RCT)	Breast ca who undergoing chemotherapy (n=240)	16 weeks of resistance and HIIT was efective in preventing increases in CRF and in reducing symptom burden for patients during chemotherapy for breast cancer. These fndings add to a growing body of evidence supporting the inclusion of structured exercise prescriptions, including HIIT, as a vital component of cancer rehabilitation	RT–HIIT-resistance training 70-80% 1RPM, 812reps, 2-3 sets followed with 3x3mins cycle ergometer RPE 16-18<br>AT–HIIT-20 mins aerobic training at moderate intensity RPE 13-15, folloerd with<br>UC-written information regarding exercise recommended by ACMS	Piper Fatigue Scale, EORTC-QLQ-C30, and Memorial Symptom Assessment Scale.	The time frame of 16 weeks is quiet longer and may affect participant engagement -no physical performance test as outcome measure not mentioned about the standard care delivered to the control group
Dunne 2016 (RCT)	colorectal liver metastasis (CRLM) (n=38)	A 4-week prehabilitation programme can deliver improvements in CPET scores and QoL before liver resection. This may impact on perioperative outcome	4-week (12 sessions) highintensity cycle, interval training programme (intensity based on 60% of VO2 peak and 90% of VO2 peak	CPET values and Quality of life (QoL) assessed using the SF36.	
Banerjee 2017 (RCT)	Bladder Ca (n=60)	Bladder cancer patients respond well to pre-surgical aerobic interval exercise, and the improvements in CRF	Vigorous intensity aerobic interval exercise ,5–10-min warmup against light resistance 70–85% MHR	Cardiorespiratory fitness (VO2peak, AT, WRpeak), feasibility, Clavien Dindo classification, LOS	The control group did not get any exercise prior to surgery

Table 1: Table summarizes the RCT study of HIIT versus MICT with the gaps

In a randomised control trial study done by Devin 2018, he found that HIIT promotes superior improvements and short-term maintenance of V˙O2peak and fat mass improvements in colorectal cancer survivors. In his study two groups were developed; Moderate Intensity Continuous Exercise (MICE) with consisted of 50 minutes of cycling at 50-70% peak heart rate, 3x/week and HighIntensity Interval Exercise (HIIE) consisted of 10 minutes warm-up at 50-70% of heart rate peak, 4minute interval, cycling at 85-95% of heart rate peak with 3 minute period of active recovery, repeated 4x, a total of 38 min/session (Devin et al. 2018). The result shows improvement in VO2 peak and reduction of fat mass in the high-intensity group. However, data regarding the patient's physical performance and quality of life was not recorded.

Other than that, short-term HIIT was feasible and safe in preoperative settings and increased cardiorespiratory fitness among the lung cancer population (Bhatia, 2019). In a study conducted by Bhatia (2019), the intervention group was given HIIT 2–3xweekly, 2 × 10-min series of cycling at peak power, measured with CPET before training,15s on-off duty cycle, 5-min warm-up, 5-min cooldown. Cardiorespiratory fitness (VO2peak), 6MWT, oxygen saturation, leg fatigue, and dyspnea (BORG scale) had been measured. However, this research did not mention about the usual care practice in the control group.

A systematic review and meta-analysis of comparative studies on HIIT in cancer prehabilitation conducted by (Palma et al. 2020) found that HIIT has potential health benefits in patients diagnosed with cancer and is feasible and safe to perform.

In another systematic review and meta-analysis by Clifford K et all 2023, the pooled results indicated several positive associations of HIIT vs standard care with CRF or postsurgical outcomes. These findings suggest that HIIT may improve patient outcomes, with robust benefits across patient populations (Clifford et al. 2023). Preoperative HIIT shows promising results and should be included in prehabilitation programs. The high degree of heterogeneity in our analysis demonstrates differences in training programs and supports the need for further well-designed studies to improve the quality of evidence and confirm effective HIIT protocols.

One of the most difficult specialities in surgery is hepatobiliary (HPB) surgery because it requires both surgical skill and a thorough understanding of pancreatic and hepatobiliary physiology. Hepatobiliary surgery shares postoperative complications with general abdominal surgery as venous

catheter-related infection, incisional infection, pulmonary atelectasis or infection, subphrenic infection, urinary tract infection, intraperitoneal haemorrhage, and gastrointestinal tract bleeding. In a systematic review and meta-analysis by Dagorno et al. (2022), the study concluded that there was no effect of prehabilitation programs in HPB surgery was observed on LOS or postoperative complications rate. However, this study did not specifically measure the exercise prehabilitation interventions effect. But, in other research, involved 1778 titles and abstracts and selected 6 (randomized controlled trial, n = 3; prospective cohort, n = 1; retrospective cohort, n = 2) that included 957 patients. Of those, 536 patients (56.0%) underwent exercise prehabilitation and 421 (44.0%) received standard care. Patients in both groups were similar with regards to important demographic factors. Prehabilitation was associated with a 5.20-day LOS reduction (P = 0.03); when outliers were removed, LOS reduction decreased to 1.85 days and was non-statistically significant (P = 0.34) which described that exercise prehabilitation may reduce LOS and morbidity following HPB surgery and studies with well-defined exercise regimens are needed to optimize exercise prehabilitation outcomes (Deprato et al 2022).

Most of the research comparing HIIT and MICT was based on pre operatively only. There are no pre and post-operative data regarding patients' physical performance and quality of life. Besides that, some research did not mention about the usual care they practice in the control group (Bhatia 2019) (Dunne 2016). While, Egegaard 2019 presents a small sample size in their research. Research done by Sara Mijwell 2018 reported the finding within the time frame of 16 weeks which is quite long and may affect participant engagement. Furthermore, the research did not measure physical performance as their outcome. While the control group did not get any exercise before surgery in research done by Banerjee 2017.

In Malaysia, there are no data regarding patient's health status such as ambulation status, performance test, and quality of life before undergoing major abdominal surgery and sequences after surgery. Besides that, there is no protocol in terms of determining the right dosage of exercise during prehabilitation phase. It is crucial to demonstrate that patients undergoing major abdominal surgery in this setting may derive benefits from a prehabilitation program. Therefore, besides the gaps of the previous studies, this study aims to evaluate the effectiveness of high-intensity interval training (HIIT)

compared to moderate intensity continuous training (MICT) in prehabilitation among patients undergoing major abdominal surgery in our setting and the sequences of the effect after operation.

1.2 Research Questions

1.21 What is the current practice amongst physiotherapists treating patients following major abdominal surgery in Malaysia?

1.22. What is the optimum prehabilitation program with a focus on exercise intervention to improve multidomain recovery following major abdominal surgery in Malaysia

1.23 How does a change in exercise intervention protocol (HIIT versus MICT) affect multidomain recovery that includes, cardiorespiratory fitness, respiratory muscle strength, physical function, pain and discomfort, psychological recovery, and health-related quality of life (HRQOL)?

1.24 What are the clinimetric properties (MCID) of selected pain, function and physical activity assessment tools used following major abdominal surgery in Malaysia

1.3. Objectives

Primary Objective

To determine the effect of HIIT over MICT on physical performance which is cardiorespiratory fitness (6MWT) and maximum inspiratory pressure among patients undergoing major abdominal surgery.

Secondary Objectives

i) To assess the effect of HIIT over MICT on physical performances (Short Physical Performance Battery and 1 minute Sit to Stand), upper limb strength (Hand Grip Test) and body composition (Bioimpedance test), fatigue (Fatigue Severity Scale), psychological recovery (the Hospital Anxiety Depression Scale (HADS) and health related quality of life (EuroQoL).

ii) To identify what is the type of exercise timing, dosing and content of prehabilitation

iii) To determine the current practice among Malaysian physiotherapists on treating patients undergoing major abdominal surgery

iv) To determine the clinometric properties of selected pain, function and physical activity assessment tools used following major abdominal surgery in Malaysia.

1.4 Hypothesis

i. There will be variations in the standard of practice to these key questions across different states in Malaysia and among Malaysian physiotherapists in treating patients undergoing major abdominal surgery.
ii. HIIT is non-inferior to MICT in improving cardiorespiratory fitness, maximum inspiratory pressure, physical performance, upper limb strength, body composition, fatigue, psychological recovery and health-related quality of life

1.5 Expected Outcome and Benefit

Findings from this study will warrant its application to every prehabilitation patient who undergoes major abdominal surgery:

i. Aerobic training, resistance training and respiratory training might help the patient to improve physical performance and quality of life before the operation.
ii. Prehabilitation exercise might help patients to recover faster by reducing the length of stay and reducing the rate of PPC and mortality.
iii. The results of the present study will help physiotherapists to extend the protocol and improvise the protocol if necessary.
iv. First RCT with robust methodology in Malaysia and worldwide
v. the outcomes of this study inform the implementation of clinical and regulatory guidelines on prehabilitation based on the Malaysian model for patient and community safety; quality of life and standards of care of individuals scheduled for MAS surgery
vi. This research could influence the prehabilitation protocol and exercise recommendations for abdominal surgery by emphasizing the importance of precise exercise dosage.

2.0 Research Methodology

2.1 Study Design

This study will be 2 phases: (i) Phase 1 will be a cross-sectional web survey will be conducted to explore the current practice amongst physiotherapists treating patients following major abdominal surgery in Malaysia hospitals and (ii) Phase 2 will be a prospective, double-blinded randomized controlled trial which aims to determine the effect of HIIT over MICT on multi-domain recovery.

Phase 1: Web Survey on Current Practices

The first phase provides critical background information. It aims to assess the current practices among physiotherapists in Malaysia regarding prehabilitation for major abdominal surgery patients. This phase helps identify the standard care approaches, frequency, type of exercises used (if any), and possible gaps in the existing practice. Understanding these current practices is essential because:

- It sets a baseline for what is already being implemented in Malaysia.
- It helps to contextualize the results of the RCT in Phase 2, as the study participants are likely to have been treated according to these existing practices.
- The data collected will inform standard care in the control arm of the RCT (moderate-intensity continuous training, MICT).

Phase 2: RCT Comparing HIIT vs. MICT

The randomized controlled trial (RCT) in Phase 2 will test the effectiveness of High-Intensity Interval Training (HIIT) compared to current standard of care (which will likely resemble the practices identified in Phase 1, i.e., MICT). This phase aims to demonstrate whether a change in the type of prehabilitation exercise improves multidomain recovery, including functional capacity, postoperative complications, and quality of life.

Link Between Phases:

Phase 1 lays the foundation for Phase 2 by providing a clearer understanding of the standard care practices that will serve as the control intervention (MICT). Without Phase 1, the RCT in Phase 2 may lack context or not fully represent the local practices in Malaysia, which may differ from international standards. Including both phases provides a comprehensive approach that not only evaluates an innovative intervention (HIIT) but also benchmarks it against real-world practices in Malaysia. In conclusion, both phases complement each other, with Phase 1 informing the setup of

Phase 2, ensuring the findings are relevant to local practice and helping to close the gap between current practice and emerging evidence-based interventions.

Phase 1 (Web Survey)

A survey form using a cross-sectional, observational design will be created and distributed via an online survey tool. We will identify a tool that can house questions designed with built-in logic and direct respondents to sections of an online web survey based on their preceding answers. The online survey will then be circulated and a reminder regarding completion of the web survey will be submitted. The surveys will provide a basic analysis of raw data. Ethics approval will be obtained from the UKM and KKM ethics committee.

All Malaysian hospitals conducting general surgery will be identified. Hospitals will be contacted to establish if abdominal surgery was performed and whether the facility provided a physiotherapy service to patients undergoing abdominal surgery. The physiotherapy head of the department, Cardiovascular Pulmonary Special Interest Group (CVPG), Malaysian Physiotherapy Association (MPA) will provide further contact details and/or email addresses of physiotherapists. Once the participants who meet the inclusion criteria decide to take part in the study, they will be required to fill in the consent form in the first section of the online tools before continuing to the following section. Participants will be encouraged to forward the email to other relevant clinicians, increasing the response rate.

Before use, 10% (n=7) independent individuals will be identified to validate the survey questionnaire for readability and face validity. The participants will be physiotherapists with a particular interest and knowledge regarding major abdominal surgery and health professionals in different fields of work. The main objective is to identify any unanticipated problems and ambiguity within the instructions and questions and recognize the time commitments required to complete the survey, allowing modification before dissemination.

Study Period

The study will be carried out in October 2024- October 2025.

Study Location

The study will be carried out through an online survey form and distributed to the entire Malaysia.

Study Population

Inclusion Criteria:

- A qualified physiotherapist who has treated patients following abdominal surgery in Malaysian hospitals.

Exclusion Criteria:

- Physiotherapists who are treating other than Cardiorespiratory patients
- Cardiorespiratory physiotherapist who is dealing with administration work with less clinical practice.

Sampling and Sample Size Calculations

Sample size calculation:

- Participants will be recruited via convenience sampling and the size estimation was calculated using the population proportion formulae.
- the percentage of cardiorespiratory physiotherapists = 113/19,135 x 100% = 0.6%
- the sample size formula for a single proportion is:

Where: z = 1.96 p

= 0.006 or 0.6%

delta = 0.02 (within 1% of the true value)

So, N= 57

Addition of 30% non-response rate, the total sample will be N= 74 physiotherapists.

$$n = \left(\frac{z}{\Delta}\right)^2 p(1-p)$$

n = number of samples
z = 1.96 (for α error 0.05, two tailed or 95% CI)
2.58 (for α error 0.01, two tailed or 99% CI)
p = proportion of disease or factor under study
Δ = width of the confidence interval

Data collection

A questionnaire was borrowed from Patman et al (2017), and Aldhuhoori et al (2021) a previous study with similar objectives completed in Australia and United Emirate Arabs. The questionnaire will be modified slightly based on current evidence with the consent of the origin to produce the final version. The questionnaire had 51 questions cutting across 7 sections that investigated the assessment tools and interventions and explored current practice amongst physiotherapists treating patients following abdominal surgery in Malaysia hospitals. The questionnaire will be divided into seven sections, including: i. participants' information, ii.patient demographics, iii.patient-physiotherapy factors for commencing treatment, iv prescription and v. dosage of interventions, vi.mobility prescription following abdominal surgery (frequency, intensity, and duration of mobility prescription), and vii. discharge planning.

The questionnaire will be validated to identify any problems with the English version web survey among a group of volunteers of 10% of physiotherapist from HTCM who has not treated other than cardiorespiratory patient. Following validation of the questionnaire, 74 participants will be invited and directed to questions in each section based on their preceding answers. Most of the questionnaire will be close-ended, incorporating a ranking system and matrix scale, with some open-ended questions to allow respondents to elaborate as appropriate. A link of the web survey will be distributed to potential participants and informed consent will be obtained upon their response. After two weeks, a reminder email will be resent again. Figure I the research flow chart for study 1.

Figure I

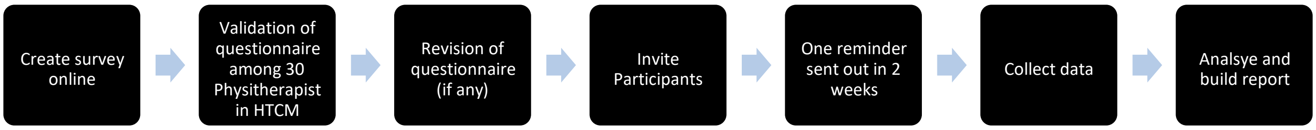

Study Method

- Prior to use, 7 (10%) independent individuals will be identified to validate the survey questionnaire for readability and face validity
- The participants will be from physiotherapists with a particular interest and knowledge regarding major abdominal surgery and health professional in a different field of work.
- The main objective to identify any unanticipated problems and ambiguity within the instructions and questions and recognize time commitments required to complete the survey, allowing modification prior to dissemination.

A questionnaire was borrowed from Patman (2017) and Aldhuhoori (2021) a previous study with almost similar objectives completed in Australia and United Arab Emirates(UAE). The questionnaire was modified slightly with the consent of the origin to produce the final version. The questionnaire had 151 questions cutting across 7 sections that investigated the assessment tools and interventions and explored current practice amongst physiotherapists treating prehabilitation patients following abdominal surgery in Malaysia hospitals. The questionnaire collected participants' information, patient demographics, patient-physiotherapy factors for commencing treatment, prescription and dosage of interventions, mobility prescription following abdominal surgery (frequency, intensity, and duration of mobility prescription), and discharge planning. The questionnaire is generally close ended, incorporating a ranking system and matrix scale.

Statistical Analysis

- Data collected from the web survey were analysed with descriptive statistics, using version 29.0 of Statistical Package for the Social Sciences (SPSS) (IBM Corporation, New York, United States of America).
- Categorical data will be analysed descriptively in terms of count, frequency and proportions, primarily reporting percentages and means, specifically clarifying the total responses (n).

- Means will be used to decipher the matrix styled questions. a 5-point likert scale will be used, aligning with “never” to “always”.
- For ranking styled questions, respondents will limit to three responses, allowing greater control of bias.

Phase 2 (Randomized Control Trial Study)

The recruitment of all 70 participants is expected to be completed by July 2026, with data collection concluding by October 2026. The study results are anticipated in November 2026, and manuscript preparation is projected for completion by December 2026.

Study Location

The study will be conducted in Hospital Canselor Tuanku Muhriz, Universiti Kebangsaan Malaysia in Cheras, Kuala Lumpur.

Study population

Inclusion criteria

- Adults aged 18 and above.
- Scheduled for major abdominal surgery.
- Able to perform the Six-Minute Walk Test (6MWT).
- Provide written informed consent.

Exclusion criteria

- Emergency operation
- Medically unstable or severe comorbidities that contraindicate exercise (e.g., unstable cardiovascular disease).
- Impaired cognition or confusion
- Impaired vision, cognition or physical impairment (UL and LL) in functional task components

Study Design

This study is a randomized control trial, and a double-blinded assessor (blinding of patients and assessors and intention to treat analysis). The study will be carried out at the Hospital Canselor Tuanku

Mukhriz (HCTM), Cheras, Kuala Lumpur, Malaysia. Participants will be recruited from the surgery and anaesthetic clinic at the HCTM. Randomization of participants by using a computer-generated random number sequence (Block Randomization), will allocate each participant to either an intervention group or standard care group (1- 70).

Blinding and allocation

Patients and outcome assessors, will remain blinded to treatment allocation. However, the treating physiotherapists cannot be blinded due to the nature of the intervention. To maintain blinding, details of the intervention will not be recorded in the medical records. Outcome assessments will be conducted by a blinded assessor based off-site from the outpatient department, ensuring impartiality in both inpatient and outpatient settings. Intervention sessions will occur on different days from those scheduled for outcome assessments to further reduce the risk of unblinding. If a participant from the intervention group discloses details of their intervention to the assessor, this will be documented and reported, with the rationale recorded. All analyses will be performed on an intention-to-treat basis once randomization is unblinded.

Following recruitment and baseline assessments, participants will be randomly assigned to one of two groups which will be control group (MICT) or the intervention group (HIIT) using a computer-generated random number sequence. Allocation will be conducted by an independent individual utilizing a block randomization method (5 blocks of 14). An administrative assistant, independent of the trial, will prepare 70 sequentially numbered opaque envelopes, each containing a group allocation. A physiotherapist, trained in the study protocol, will deliver the intervention. Participants in the control group will receive moderate-intensity continuous training (MICT), while those in the intervention group will engage in high-intensity interval training (HIIT) preoperatively. Both groups will commence and conclude sessions with a warm-up and cool down to mitigate the risk of adverse events.

This study is will be conducted at a teaching hospital of the Hospital Canselor Tuanku Muhriz, Malaysia. Prospective consecutive patients will be invited to participate in the study prior to elective major abdominal surgery from February 2025 to July 2026. Fig 2 summarises the design of the trial, and each of the trial's aspect is described in detail below.

Sampling and Size Calculation

The sample size was calculated based on the primary outcome measure (6MWT). A recent systematic review of prehabilitation in patients undergoing major abdominal surgery reported significant improvement in 6MWT, with a mean difference of 29.4 meters (95% CI: 5.6 to 53.3 meters; $p = 0.02$) compared to standard care [31]. The estimated standard deviation was approximately 101.6 meters, corresponding to an effect size (Cohen's *d*) of 0.29. Using this effect size, with α of 0.05 and power of 80%, the required sample size was calculated to be 54 participants. Allowing for a 30% dropout rate, a total of 70 participants (35 per group) will be recruited. Sample size calculation was performed using G*Power version 3.1.9.7.

Study Method

Intervention care group (HIIT)

Participants in the intervention group will receive the same care as the standard care group. Additionally, for aerobic exercise this will be a moderate intensity which is 55-75% of maximum heart rate (MHR) in week one, with graduation to high intensity in week two on > 80% of MHR [24]. Aerobic exercise training (eg: Treadmill, Cycle ergometer, body weight exercises) 1-3 times per week (30 minutes) for an overall duration typically 2-4 weeks prior to the date of surgery. Patients' Maximum Heart rate (210-age) and Karvonen Formula will be used for the targeted heart rate. Patients' heart rate will be monitored along the aerobic training using a smartwatch. However, those patients who are on beta-blockers or with underlying cardiac conditions, training intensity will be guided by the Rating of Perceived Exertion (RPE) between 16-18 RPE score, with appropriate adjustments made to ensure safety. The interval training will be 30-60 seconds of high-intensity training with 1- 2 minutes of active recovery (light activity or complete rest) between intervals to facilitate recovery. This will be repeated for a total of 5-10 cycles [32].

In addition to aerobic training, participants will receive the same progressive resistance exercise and inspiratory muscle training, with intensity matched to that of the standard care of

MICT group. Aim for a total session duration of about 20-30 minutes, including warm-up and cool-down. This includes 10-15 minutes of HIIT, depending on the patient's tolerance. Sessions will be done 1-3 times per week, with rest days in between to allow for recovery. Participants will also receive an illustrated handout to guide continued training support by caregivers, including recommendations on maintaining a standard amount of aerobic and strength exercise per week. A detailed session attendance checklist and vital signs will be recorded at each session to monitor adherence.

All other aspects of patient care such as preoperative management, general anaesthesia, intraoperative ventilation settings, fluid administration, prophylactic antibiotic use, pain management, management of lines and drains, general nursing care, and discharge planning will be administered at the discretion of nurses and physicians in accordance with routine clinical practices at hospital for both groups.

Standard Care for Control Group (MICT)

This will be a tailored and supervised by a single physiotherapist (more than 10 years clinical experiences in cardiorespiratory) in outpatient hospital setting. Participants will engage in an aerobic (moderate intensity continuous training), strengthening exercise and inspiratory muscles training program prior to the date of their surgery.

For aerobic exercise this will be a low intensity in week one with graduation to moderate intensity. Moderate intensity continuous training (MICT) will allow 55-75% of maximum heart rate (MHR) [33]. Aerobic exercise training (eg: treadmill, cycling, walking exercise) 1-3 times per week (60 minutes) for overall duration typical 2-4 week prior to date of surgery. Patients' Maximum Heart rate (210-age) and Karvonen Formula will be used for targeted heart rate. Patients' heart rate will be monitored along the aerobic training using smart watch. However, the Karvonen formula may not be suitable for all patient populations, particularly those on beta-blockers or with underlying cardiac conditions. In such cases, training intensity will be guided the RPE between 10-13 RPE score, and appropriate modifications will be made to ensure safety. This approach allows for individualized intensity adjustment while maintaining the safety and effectiveness of the intervention.

For the progressive resistance exercise, the following muscle groups will be targeted: biceps brachii, triceps brachii, pectoralis major and minor, latissimus dorsi, deltoid and rhomboids using free weights. The intensity will be 70-80% of estimated 10 repetitions

maximum. The load not provided joint pain or severe muscle fatigue and/or arm fatigue on RPE of 10-13 on 6-20 RPE score [60]. Aim for a total session duration of about 45-60 minutes, including warm-up and cool-down. This includes 30-45 minutes of MICT, depending on the patient's tolerance. Sessions will be done 1-3 times per week [60].

In addition to the PVC weighted bar, we also used different types of resistance training such as band, dumbbells, kettlebells. Inspiratory muscles training will be given 20 mins per day with 30% of maximum inspiratory pressure (MIP) [14].

Participants will also be given an illustrated handout to assist further training support by the carer (advice regarding maintaining a set amount of aerobic/strength exercise per week standard [1][2][14]. Detailed session attendance checklist and vital sign will be recorded for every attendance for adherence.

	**HIIT**	**MICT**
Duration and Intensity	10- 15 minutes<br><br>Alternating short bursts of high-intensity exercise (>80% of peak heart rate) with periods of low-intensity recovery.	30-45 minutes<br><br>Continuous moderate-intensity exercise (55-75% of peak heart rate).
Borg's RPE	16-18	10-13
Borg's dyspnea	5-8	3-4
Talk test	Difficult to speak without pausing a breath every few words	Able to speak in full sentence
Type of exercise	Start warm-up for 5-10 minutes.<br>Jogging and higher speed cycling.	Walking, cycling, stair climbing

Table 2: The table shows the differences between HIIT and MICT that will be conducted in our setting

Flowchart for Patient Recruitment and Screening

1. Start - Begin the recruitment and screening process.

2. Patient Eligibility Screening

Step 1: Check if the patient is awaiting major abdominal surgery.

Yes: Proceed to the next step.

No: Exclude from study.

Step 2: Check if patients Medically unstable or severe comorbidities that contraindicate exercise (e.g., unstable cardiovascular disease), Impaired cognition or confusion and impaired vision, cognition or physical impairment (UL and LL) in functional task components

Step 3: Conduct additional baseline assessments (e.g., medical history, strength, flexibility, respiratory function) for patients included in the study.

Step 4: Obtain informed consent from eligible patients.

Step 5: Randomize patients into the HIIT or MICT intervention groups.

End -Complete the screening and recruitment process.

Data collection

Demographic data and preoperative, intraoperative, and postoperative variables will be collected from participants and their medical records. Baseline assessments will be conducted 4 (± 1) weeks in outpatient setting following referral for exercise based prehabilitation prior to date of surgery. Follow up assessment will continue to 2 days (±1) before operation in the inpatient setting, prior to discharge, 4 -weeks, and 12-week post operatively. These follow-ups will take place in the research room within the physiotherapy unit.

All measurements will be conducted by an independent, trained assessor located off-site, who will remain blinded to group allocation. Face-to-face administration of follow-up tests and questionnaires will be performed by outcome assessors at four time points: preoperatively, prior to discharge, 4 weeks, and 3 months postoperatively, to maintain consistency across participants.

Post-hospital discharge follow-ups will be contacted via phone. Participants who cannot be reached by phone for 14 consecutive days from the scheduled assessment date will be considered lost to follow-up for post-discharge outcomes.

Primary outcome measures

The 6-Minute Walk Test (6MWT)

The 6MWT is a widely used measure to assess the submaximal level of functional capacity [34]. The test involves walking back and forth along a 30-meter indoor track for as many laps as possible within a 6-minute period [35,36]. Following the American Thoracic Society (ATS) guidelines, the test will be conducted before initiating prehabilitation, with the better result from two attempts recorded as the baseline measurement [37]. Participants will be informed of the elapsed time at the end of each minute during the test, but no additional encouragement will be provided [37].

Maximal Inspiratory Pressure

Inspiratory muscle strength will be assessed by measuring the maximum inspiratory pressure (MIP) generated at the mouth using the PowerBreathe K2 device. Patients will be seated in an upright position and instructed to exhale fully to their residual volume before performing a maximal and rapid inspiratory effort lasting 3 seconds. The highest 1-second average value from each trial will be recorded. To ensure reliability, three consistent measurements (defined as having a coefficient of variation <10%) will be selected from 5 to 8 maximal trials, with each trial separated by a 60-second rest interval [38].

Secondary outcome measures

1 Minute Sit to Stand Test (1MSTST)

The 1MSTST is recognized as a tool for assessing functional status and predicting fall risk. It has shown a good correlation with exercise capacity in patients with chronic obstructive pulmonary disease (COPD) [39]. Studies have demonstrated a significant correlation between the number of repetitions completed in the 1MSTST and essential functional outcomes in patients with COPD, including the 6-minute walk distance (6MWD), quadriceps muscle strength, and physical activity levels [39, 40, 41]. These findings suggest that the 1MSTST may serve as a valid measure of exercise capacity following respiratory rehabilitation, especially in

populations with comorbid of pulmonary complications, which is common in patients with abdominal surgery.

Short Physical Performance Battery (SPPB)

The SPPB will be used to assess lower extremity physical performance status. It is an objective outcome measures of balance, lower extremity strength, and functional capacity which includes three domains (walking, sit-to-stand and balance) [42]. SPPB had an excellent interrater reliability (ICC = 0.92) among people with respiratory condition [43]. Besides, it may serve as a reliable surrogate endpoint for all-cause mortality in clinical trials aiming to evaluate the efficacy of specific treatments or rehabilitation programs in improving health outcomes [44].

The Hospital Anxiety and Depression Scale

Anxiety and depression will be measured using the 14-item Hospital Anxiety and Depression Scale (HADS), which comprises two subscales: one for anxiety (HADS-A) and one for depression (HADS-D), each containing seven items [45]. Each item is scored on a 4-point Likert scale ranging from 0 (not present) to 3 (maximum), with a maximum subscale score of 21 points [46]. A cut-off score of $\geq$8 points will be applied to identify cases of anxiety or depression, as this threshold has been shown to optimize sensitivity and specificity for both subscales [46]. The HADS is a validated and reliable instrument, with Cronbach's α values of 0.81 for HADS-A and 0.88 for HADS-D [46,47]. It has been widely translated and extensively utilized in international research [46,48], demonstrating high internal consistency in inpatient populations, including those undergoing median sternotomy [48].

Hand grip strength

Hand grip strength will be measured in kilogram using a hand-held dynamometer with three attempts to assess physical fitness that related to muscular strength. The peak pressure of each 5-second attempt and the highest value will be recorded [49]. This hand-held dynamometer has excellent reliability (ICC = 0.98) and construct validity of r = 0.99 (very high correlation) with Rolyan dynamometers [50].

EuroQol (EQ-5D-5L)

Health related quality of life will be evaluated using self-assessment questionnaire EuroQol EQ-5D-5L. It consisted of five component scales, namely mobility, self-care, usual activities, pain, and anxiety. Recent evidence suggested that the newer 5-level version of the EQ-

5D had improved measurement properties, including feasibility, ceiling effects, sensitivity, and convergent validity [51]. The EQ-5D-5L had good test-retest reliability, as indicated by intraclass correlation coefficients (ICCs) ranging from 0.65 to 0.91 for the EQ-5D index [52].

Self-Assessment of Physical Activity Questionnaire (SAQ)

Self-Assessment of Physical Activity Questionnaire (SAQ) will be used for physical capacity. The SAQ is a 13-item, self-administered activity questionnaire assessing common physical activities associated with personal care, ambulation, household tasks and recreation [53]. All these activities have a known metabolic equivalent (MET) obtained from the Compendium of Physical Activities. Each question required a 'yes' or 'no' response. A SAQ score will be obtained by recording the corresponding MET value of the most demanding activity that the participant perceived they could complete without symptoms [53]. All scores will be correlated against measured of peak oxygen consumption [53].

Fatigue Severity Scale (FSS)

The Fatigue Severity Scale (FSS) is a method of evaluating the impact of fatigue. The Fatigue Severity Scale (FSS) is widely used to assess the impact of fatigue on daily functioning. Its psychometric properties have been evaluated in various populations, demonstrating its reliability and validity [54]. The FSS is a short questionnaire that requires patients to rate their level of fatigue. The FSS questionnaire contains nine statements that rate the severity of fatigue symptoms. Read each statement and circle a number from 1 to 7, based on how accurately it reflects the condition during the past week and the extent to which patients agree or disagree that the statement applies to patients [55].

The Global Rating of Change Scale

This is a self-reported measure of patients' perceived change. It will be administered prior to the performance-based assessments preoperatively, prior to discharge, 4 weeks and 3 months postoperatively. Participants will be asked: 'Overall, how do your arms function now, compared with how your arms functioned at the initial assessment before surgery?' Responses will be recorded according to a 7-point scale from 'very much improved' to 'very much worse'. It has been previously reported that when participants rate their change as 'minimally improved', 'no change' or 'minimally worse', it is unlikely that a clinically important difference has occurred;

therefore, these patients are grouped into an 'unchanged' category [56]. Responses of 'much worse', 'very much worse', 'much improved' and 'very much improved' indicate a clinically important difference has occurred and therefore these patients are grouped into a 'changed' category [56].

Bio-electrical Impedance Analysis

An InBody device utilizing Bio-electrical Impedance Analysis will be used to assess body composition by estimating key components such as lean body mass, fat mass, and total body water. This method involves passing a low-level electrical current through the body and measuring the resulting impedance to estimate body composition parameters, including total body water, extracellular water, intracellular water, fat mass, fat-free mass, body fat percentage, and bone mineral content [57].

Bioelectrical Impedance Analysis has been validated as a reliable method for assessing body composition, showing strong correlations with CT-derived measurements of torso fat volume and waist fat area, with correlation coefficients ranged from 0.86 to 0.95 in both men and women ($p < 0.001$) [58]. InBody devices showed a 98% correlation with dual-energy X-ray absorptiometry (DXA), with strong agreement across all body mass index (BMI) categories [59]

Adherence monitoring

Data related to exercise prescription, progression, complaints, injuries, and symptoms during training sessions for the both groups will be systematically recorded. Participants will be encouraged to adhere to the exercise guidelines outlined in their weekly flyers. Attendance will be documented, including the total number of sessions attended and successfully completed. Detailed reasons for incomplete sessions or participant dropouts will be recorded to evaluate non-compliance. Adherence to the prehabilitation exercise program will be closely monitored, alongside compliance with the prescribed exercise regimen. Additionally, participants will be asked to report the duration of their adherence and rate their adherence to home-based activities using a numerical scale [60].

Duration and timeline

The recruitment of all 70 participants is expected to be completed by July 2026, with data collection concluding by October 2026. The study results are anticipated in November 2026, and manuscript preparation is projected for completion by December 2026. The final manuscript will

be prepared following the CONSORT extension guidelines for pragmatic trials involving non-pharmacological interventions, as outlined in Fig 1.

Statistical analysis

The analysis will follow the intention-to-treat, whereby all participants will be analyzed according to the groups to which they were originally randomized, regardless of their adherence to the intervention protocol. SPSS version 27.0 will be used to analyse all collected data. Missing data will be handled using multiple imputation, which creates several datasets by statistically estimating missing values and then combines the results to account for uncertainty. This method is preferred over last observation carried forward, as it reduces bias and more accurately reflects variability in clinical studies [61].

Descriptive statistics will summarize participants' baseline demographic and clinical characteristics. Continuous variables will be reported as mean (standard deviation, SD) or median (interquartile range, IQR) depending on the distribution, while categorical variables will be reported as frequency (percentage). Baseline comparability analysis of the clinical outcomes between groups will be performed using independent *t*-tests for continuous variables and Chi-square tests for categorical variables.

The primary outcomes (6MWT and MIP) and secondary outcomes (1MSTS, SPPB, hand grip strength, FSS, Bio-impedance Test, EuroQol, SAQ and Global Rating of Change Scale) will be measured at multiple time points: baseline, post-prehabilitation (4 weeks), before hospital discharge, and at 4 and 12 weeks postoperatively. To evaluate changes over time and between-group differences, a linear mixed-effects model (mixed model ANOVA) will be employed, provided the assumptions of normality and sphericity are met. Normality will be evaluated using the Kolmogorov–Smirnov test, while sphericity will be assessed using Mauchly's test, Mixed model ANOVA will provide results for time effect, group effect, and time-group interaction effect. Statistically significant time effect and group effect indicates significant within-group changes and between-group difference respectively, while significant time-group interaction effect determine if the interventions yield the desired effect on the dependent variables. Post hoc pairwise comparisons will be conducted using Bonferroni correction where appropriate.

The level of significance is set at $p<0.05$. Effect sizes will be determined using Cohen's d, with values of 0.2, 0.5, and 0.8 indicating small, medium, and large effects, respectively [62]. Additional logistic regression will be used to determine preoperative, perioperative and postoperative risk factors associated with the development of respiratory complications. This will be an exploratory analysis, which may identify trends of predictors reported in the literature having an individual effect on postoperative respiratory complications. For all tests conducted, a p value of <0.05 (two-sided) will be considered statistically significant, and mean differences (95% CI) will be reported. The results of this study will be reported in accordance with the CONSORT 2010 guidelines.

	STUDY PERIOD						
	Enrolment & allocation	Baseline	Intervention	Post-intervention assessment	Follow up	Follow up	Follow up
**TIMEPOINT**	February 2025- July 2026	3-5 week before operation	2- 4 weeks	Preoperatively	Post-operatively prior to discharge	4-6 weeks post-operatively	3 months post operatively
Eligibility screen	X						
Informed consent	X						
Allocation	X						
**INTERVENTIONS**							
Intervention A (Interventional)			X				
Intervention B (control)			X				
**ASSESSMENT**							
**Sosiodemography**							
Age		X					
gender		X					
Education		X					
Occupation		X					
**Primary Measure**							
6 Minutes Walking Test (6MWD)		X		X	X	X	X
Maximal Inspiratory Pressure (MIP)		X		X	X	X	X
**Secondary Measure**							
Short Physical Performance Battery (SPPB)		X		X	X	X	X
1 Minute Sit to Stand (1MSTS)				X	X	X	X
Hand grip Test		X		X	X	X	X
Bio-impedance Test		X		X	X	X	X
Fatigue Severity Scale (FSS)		X		X	X	X	X
the Hospital Anxiety Depression Scale (HADS)		X		X	X	X	X
Euro QoL (EQ-5D-5L)		X		X	X	X	X
Self-Administered Physical Activity Questionnaire (SAQ)		X		X	X	X	X
Global Rating of Change (GRC)		X		X	X	X	X

Fig. 1 SPIRIT flow diagram for the schedule of enrolment, interventions, and assessments.

3.0 Ethical Issue

3.1 Ethics of study

Ethics approval for the study will be obtained from the UKM ethics approval committee. The study will be conducted in compliance with the principles outlined in the Declaration of Helsinki and Malaysia's Good Clinical Practice Guideline.

3.2 Informed consent

If a patient meets the above inclusion criteria, they will be invited to participate in the study by the researcher team who will provide them with an information sheet detailing the study aims and time commitments. Once a potential participant provides informed consent, they will be enrolled to participate in the study. Participants can choose to withdraw at any time.

3.3 Data Management and Quality

Data will be managed using an Excel database and SPSS version 27.0. Thorough training will be provided to all personnel involved in data collection, entry, and verification to ensure accuracy and consistency across measurements and to identify missing data. Regular checks will be performed to detect any inconsistencies within and between datasets. Data entry, security, and storage will follow strict protocols to ensure quality and confidentiality. All data will be encrypted and stored in a password-protected system with limited access, complying with privacy regulations. Additionally, two independent clinical members will serve in an advisory role to the clinical investigators, overseeing participant withdrawals, monitoring ethical conduct, and reviewing any serious adverse events.

Access to the final trial dataset will be limited to the principal investigator and designated members of the research team. There are no contractual agreements restricting the investigators' access to the data, and neither the sponsor nor the funder will have access to the raw dataset. This ensures the research team's full independence in data management, analysis, and reporting. The complete trial protocol is publicly available via the ANZCTR registry, and the raw dataset will be made available upon reasonable request and approval by the principal investigator. Process evaluation findings will be published in peer-reviewed journals and presented at relevant academic conferences.

3.4 Privacy and Confidentiality

Subject's names will be kept on a password-protected database and will be linked only with a study identification number for this research. The identification number instead of patient identifiers will be used on subject data sheets. All data will be entered into a computer that is password-protected. On completion of the study, data in the computer will be copied to the hard drive and the data in the computer erased. Hard drive and any hardcopy data will be stored in a locked office of the investigators and maintained for a minimum of three years after the completion of the study. The hard drive and data will be destroyed after that period of storage. Subjects will not be allowed to view their personal study data, as the data will be consolidated into a database.

3.4 Conflict of interest

The investigators declare they have no conflict of interest.

3.5 Publication policy

No personal information will be disclosed and subjects will not be identified when the findings of the survey are published.

3.6 Termination of study

Not applicable

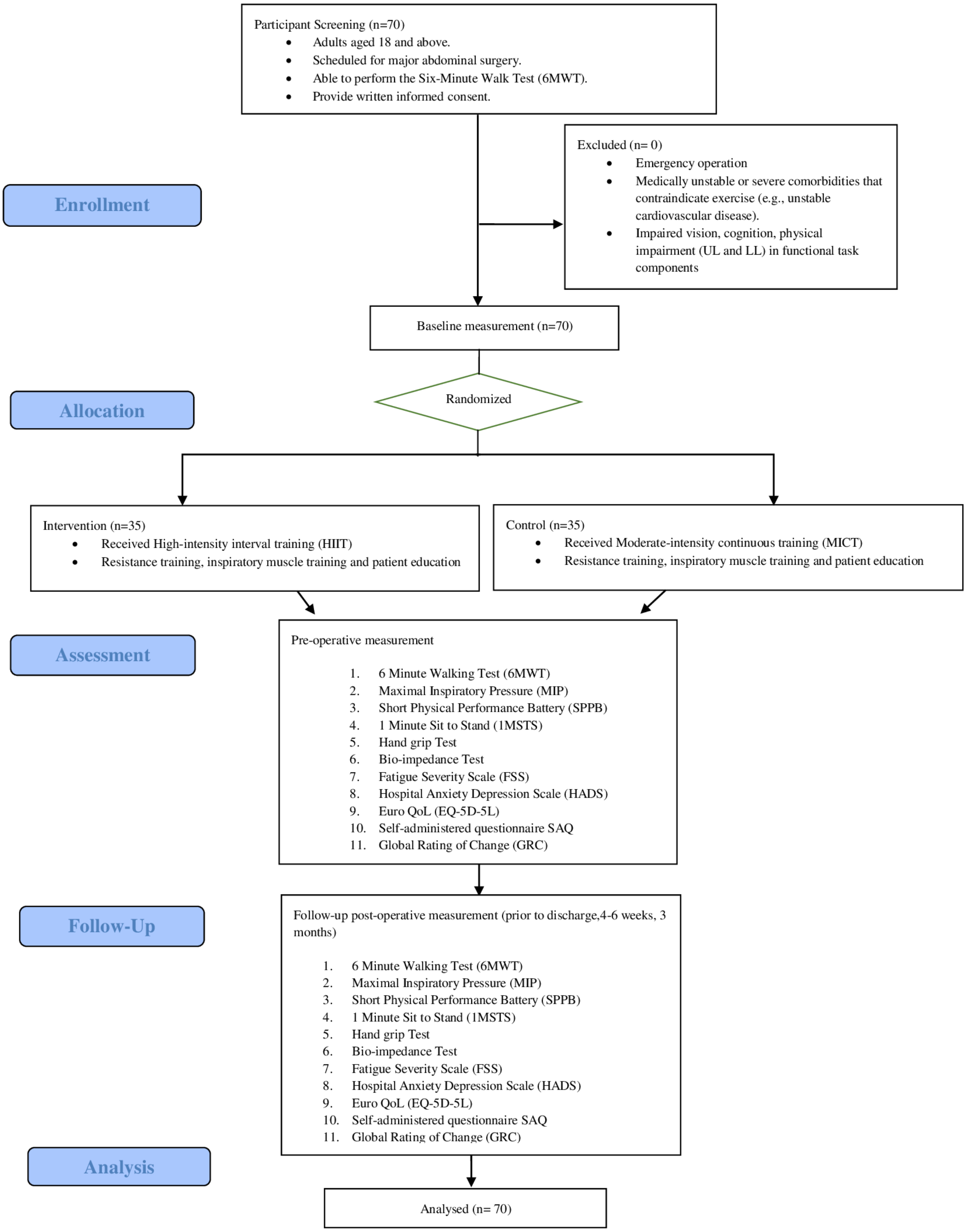

Figure 2 The participants consort diagram for Study 2

Gantt Chart

	PREPARATION FOR PROPOSAL	PREPARATION FOR PROPOSAL PRESENTATION	APPLICATION OF ETHIC COMMITTEE	DATA COLLECTION	DATA ANALYSIS	REPORT WRITING	THESIS SUBMISSION	THESIS PRESENTATION
Nov 23								
Dec 23								
Jan 24								
Feb 24								
Mar 24								
Apr 24								
May 24								
Jun 24								
July 24								
Aug 24								
Sept 24								
Oct 24								
Nov 24								
Dec 24								
Jan 25								
Feb 25								
Mar 25								
Apr 25								
May 25								
Jun 25								
July 25								
Aug 25								
Sept 25								
Oct 25								
Nov 25								
Dec 25								
Jan 26								
Feb 26								
Mar 26								
Apr 26								
May 26								
Jun 26								
July 26								

**Aug 26**								
**Sept 26**								
**Oct 26**								
**Nov 26**								
**Dec 26**								

Reference

1. Boden I. Physiotherapy management of major abdominal surgery. J Physiother. 2024; 70: 170-180. doi:10.1016/j.jphys.2024.06.005. PMID: 38902197
2. Boden I, Skinner EH, Browning L, et al. Preoperative physiotherapy for the prevention of respiratory complications after major abdominal surgery: pragmatic, double blinded, multicentre randomised controlled trial. Br Med J (Clin Res Ed). 2018; 360: j5916. doi:10.1136/bmj.j5916. PMID: 29367198
3. Boden I, Reeve J, Robertson IK, et al. Effects of preoperative physiotherapy on signs and symptoms of pulmonary collapse and infection after major abdominal surgery: secondary analysis of the LIPPSMAck-POP multicentre randomised controlled trial. Perioper Med (Lond). 2021; 10: 36. doi:10.1186/s13741-021-00206-3. PMID: 34689825
4. Haines KJ, Skinner EH, Berney S, Austin Health POST Study Investigators. Association of postoperative pulmonary complications with delayed mobilisation following major abdominal surgery: an observational cohort study. Physiotherapy. 2013; 99: 119-125. doi:10.1016/j.physio.2012.05.013. PMID: 23219632
5. Piccioni F, Spagnesi L, Pelosi P, et al. Postoperative pulmonary complications and mortality after major abdominal surgery. An observational multicenter prospective study. Minerva Anestesiol. 2023; 89: 964-976. doi:10.23736/S0375-9393.23.17382-2. PMID: 37671537
6. Fernandez-Bustamante A, Frendl G, Sprung J, et al. Postoperative pulmonary complications, early mortality, and hospital stay following noncardiothoracic surgery: a multicenter study by the perioperative research network investigators. JAMA Surg. 2017; 152: 157-166. doi:10.1001/jamasurg.2016.4065. PMID: 27829093

7. Brown SR, Mathew R, Keding A, et al. The impact of postoperative complications on long-term quality of life after curative colorectal cancer surgery. Ann Surg. 2014; 259: 916-923. doi:10.1097/SLA.0000000000000407. PMID: 24374539
8. Shander A, Fleisher LA, Barie PS, et al. Clinical and economic burden of postoperative pulmonary complications: patient safety summit on definition, risk-reducing interventions, and preventive strategies. Crit Care Med. 2011; 39: 2163-2172. doi:10.1097/CCM.0b013e31821f0522. PMID: 21572323
9. Altman AD, Helpman L, McGee J, et al. Enhanced recovery after surgery: implementing a new standard of surgical care. CMAJ. 2019; 191: E469-E475. doi:10.1503/cmaj.180635. PMID: 31036609
10. Melloul E, Lassen K, Roulin D, et al. Guidelines for Perioperative Care for Pancreatoduodenectomy: Enhanced Recovery After Surgery (ERAS) Recommendations 2019. World J Surg. 2020; 44(7) :2056-2084. doi: 10.1007/s00268-020-05462-w. PMID: 32161987
11. Kehlet H, Wilmore DW. Evidence-based surgical care and the evolution of fast-track surgery. Ann Surg. 2008; 248: 189-198. doi:10.1097/SLA.0b013e31817f2c1a. PMID: 18650627
12. Paton F, Chambers D, Wilson P, et al. Effectiveness and implementation of enhanced recovery after surgery programmes: a rapid evidence synthesis. BMJ Open. 2014; 4: e005015. doi:10.1136/bmjopen-2014-005015. PMID: 25052168
13. Kocián P, Pazdírek F, Přikryl P, et al. Should minimally invasive approaches in rectal surgery be regarded as a key element of modern enhanced recovery perioperative care? Acta Chir Belg. 2023; 123: 163-169. doi: 10.1080/00015458.2021.1971871. PMID: 34423745

14. Boden I, Denehy L. Respiratory prehabilitation for the prevention of postoperative pulmonary complications after major surgery. Curr Anesthes Reports. 2022; 15: 1-5. doi:10.1007/s40140-021-00495.

15. Boden I, Reeve J, Jernås A, et al. Preoperative physiotherapy prevents postoperative pulmonary complications after major abdominal surgery: a meta-analysis of individual patient data. J Physiother. 2024; 70: 216-223. doi: 10.1016/j.jphys.2024.02.012. PMID: 38472053

16. Carli F, Charlebois P, Stein B, et al. Randomized clinical trial of prehabilitation in colorectal surgery. Br J Surg. 2010; 97: 1187-1197. doi: 10.1002/bjs.7102. PMID: 20602503

17. Hoogeboom TJ, Dronkers JJ, Hulzebos EH, van Meeteren NL. Merits of exercise therapy before and after major surgery. Curr Opin Anaesthesiol. 2014; 27: 161-166. doi: 10.1097/ACO.0000000000000062. PMID: 24500337

18. Scott MJ, Baldini G, Fearon KC, et al. Enhanced Recovery After Surgery (ERAS) for gastrointestinal surgery, part 1: pathophysiological considerations. Acta Anaesthesiol Scand. 2015; 59(10): 1212-1231. doi: 10.1111/aas.12601. PMID: 26346577

19. Moran J, Guinan E, McCormick P, et al. The ability of prehabilitation to influence postoperative outcome after intra-abdominal operation: a systematic review and meta-analysis. Surgery. 2016; 160: 1189-1201. doi: 10.1016/j.surg.2016.05.014. PMID: 27397681

20. Weiner P, Inzelberg R, Davidovich A, et al. Respiratory muscle performance and the Perception of dyspnea in Parkinson’s disease. Can J Neurol Sci. 2002; 29: 68-72. doi:10.1017/s031716710000175x. PMID: 11858539

21. Barberan-Garcia A, Vogiatzis I, Solberg HS, et al. Effects and barriers to deployment of telehealth wellness programs for chronic patients across 3 European countries. Respir Med. 2014; 108: 628-637. doi: 10.1016/j.rmed.2013.12.006. PMID: 24451438
22. Weston M, Weston KL, Prentis JM, Snowden CP. High-intensity interval training (HIT) for effective and time-efficient pre-surgical exercise interventions. Perioper Med (Lond). 2016; 5: 2. doi:10.1186/s13741-015-0026-8. PMID: 26770671
23. Guiraud T, Nigam A, Gremeaux V, et al. High-intensity interval training in cardiac rehabilitation. Sports Med. 2012; 42: 587-605. doi:10.2165/11631910-000000000-00000. PMID: 31543179
24. Clifford K, Woodfield JC, Tait W, et al. Association of preoperative high-intensity interval training with cardiorespiratory fitness and postoperative outcomes among adults undergoing major surgery: A systematic review and meta-analysis. JAMA Netw Open. 2023; 6: e2320527. doi:10.1001/jamanetworkopen.2023.20527. PMID: 37389875
25. Atakan MM, Li Y, Koşar ŞN, et al. Evidence-based effects of high-intensity interval training on exercise capacity and health: a review with historical perspective. Int J Environ Res Public Health. 2021; 18: 7201. doi:10.3390/ijerph18137201. PMID: 34281138
26. Bishop DJ, Botella J, Genders AJ et al. High-intensity exercise and mitochondrial biogenesis: current controversies and future research directions. Physiology (Bethesda). 2019; 34: 56-70. doi:10.1152/physiol.00038.2018. PMID: 30540234
27. Gibala MJ, Little JP, Macdonald MJ, Hawley JA. Physiological adaptations to low-volume, high-intensity interval training in health and disease. J Physiol. 2012; 590: 1077-1084. doi:10.1113/jphysiol.2011.224725. PMID: 22289907

28. Weston KS, Wisløff U, Coombes JS. High-intensity interval training in patients with lifestyle-induced cardiometabolic disease: a systematic review and meta-analysis. Br J Sports Med. 2014; 48: 1227-1234. doi:10.1136/bjsports-2013-092576. PMID: 24144531
29. Chan A-W, Tetzlaff JM, Gøtzsche PC, et al. SPIRIT 2013 explanation and elaboration: guidance for protocols of clinical trials. BMJ. 2013; 346: e7586. doi:10.1136/bmj.e7586. PMID: 23303884
30. Yamato TP, Maher CG, Saragiotto BT, et al. How completely are physiotherapy interventions described in reports of randomised trials? Physiotherapy. 2016; 102: 121-126. doi:10.1016/j.physio.2016.03.001. PMID: 27033780
31. Bohannon RW, Crouch R. Minimal clinically important difference for change in 6-minute walk test distance of adults with pathology: a systematic review. J Eval Clin Pract. 2017; 23(2): 377-381. doi: 10.1111/jep.12629. PMID: 27592691
32. Tew GA, Leighton D, Carpenter R et al. High-intensity interval training and moderate-intensity continuous training in adults with Crohn's disease: a pilot randomised controlled trial. BMC Gastroenterol. 2019; 19(1):19. :doi.org/10.1186/s12876-019-0936-x. PMID: 30696423
33. Coswig VS, Barbalho M, Raiol R, et al. Effects of high vs moderate-intensity intermittent training on functionality, resting heart rate and blood pressure of elderly women. J Transl Med. 2020; 18(1): 88. doi.org/10.1186/s12967-020-02261-8. PMID: 32066460
34. Fiorina C, Vizzardi E, Lorusso R, et al. The 6-min walking test early after cardiac surgery. Reference values and the effects of rehabilitation programme. Eur J Cardiothorac Surg. 2007; 32: 724-729. doi:10.1016/j.ejcts.2007.08.013. PMID: 17881241

35. Gallagher R, Thomas E, Astley C, et al. Cardiac rehabilitation quality in Australia: proposed national indicators for field-testing. Heart Lung Circ. 2020; 29: 1273-1277. doi:10.1016/j.hlc.2020.02.014. PMID: 32402727
36. West RR, Jones DA, Henderson AH. Rehabilitation after myocardial infarction trial (RAMIT): multi-centre randomised controlled trial of comprehensive cardiac rehabilitation in patients following acute myocardial infarction. Heart. 2012; 98: 637-644. doi: 10.1136/heartjnl-2011-300302. PMID: 22194152
37. American Thoracic Society. American College of Chest Physicians. ATS/ACCP statement on cardiopulmonary exercise testing. Am J Respir Crit Care Med. 2003; 167: 211-277. doi:10.1164/rccm.167.2.211. PMID: 12524257
38. Macías-Valle A, Rodríguez-López C, González-Senac NM, et al. Exercise effects on functional capacity and quality of life in older patients with colorectal cancer: study protocol for the ECOOL randomized controlled trial. BMC Geriatr. 2023; 23: 314. doi:10.1186/s12877-023-04026-6. PMID: 37211611
39. Reychler G, Boucard E, Peran L, et al. One minute sit-to-stand test is an alternative to 6MWT to measure functional exercise performance in COPD patients. Clin Respir J. 2018; 12(3): 1247-1256. doi: 10.1111/crj.12658. PMID: 28621019
40. Zanini A, Aiello M, Cherubino F, et al. The one repetition maximum test and the sit-to-stand test in the assessment of a specific pulmonary rehabilitation program on peripheral muscle strength in COPD patients. Int J COPD. 2015; 10: 2423–2430. doi: 10.2147/COPD.S91176. PMID: 26648705
41. Crook S, Büsching G, Schultz K, et al. A multicentre validation of the 1-Min Sit-to-Stand Test in patients with COPD. Eur Respir J. 2017; 49(3): 1601871. doi: 10.1183/13993003.01871-2016. PMID: 28254766

42. Cassidy B, Arena S. The Short Physical Performance Battery as a predictor of functional decline. Home Healthc Now. 2022; 40(3): 168-169. doi.org/10.1097/NHH.0000000000001070. PMID: 35510973

43. Medina-Mirapeix F, Bernabeu-Mora R, Llamazares-Herrán E, et al. Interobserver reliability of Peripheral Muscle Strength Tests and Short Physical Performance Battery in patients with chronic obstructive pulmonary disease: a prospective observational study. Arch Phys Med Rehabil. 2016; 97(11): 2002-2005. doi.org/10.1016/j.apmr.2016.05.004. PMID: 27255806

44. Pavasini R, Guralnik J, Brown JC, et al. Short Physical Performance Battery and all-cause mortality: systematic review and meta-analysis. BMC Med. 2016; 14(1): 215. doi: 10.1186/s12916-016-0763-7. PMID: 28003033

45. Zigmond AS, Snaith RP. The hospital anxiety and depression scale. Acta Psychiatr Scand. 1983; 67: 361-370. doi:10.1111/j.1600-0447.1983.tb09716. PMID: 6880820

46. Bjelland I, Dahl AA, Haug TT, Neckelmann D. The validity of the hospital anxiety and depression scale – an updated literature review. J Psychosom Res. 2002; 52: 69-77. doi:10.1016/s0022-3999(01)00296-3. PMID: 11832252

47. Lie I, Arnesen H, Sandvik L, Hamilton G, Bunch EH. Effects of a home-based intervention program on anxiety and depression 6 months after coronary artery bypass grafting: a randomised controlled trial. J Psychosom Res. 2007; 62: 411-418. doi:10.1016/j.jpsychores.2006.11.010. PMID: 17383492

48. Osborne RH, Elsworth GR, Sprangers MA, Oort FJ, Hopper JL. The value of the hospital anxiety and depression scale (HADS) for comparing women with early onset breast cancer with population-based reference women. Qual Life Res. 2004; 13: 191-206. doi:10.1023/B:QURE.0000015292.56268.e7. PMID: 15058800

49. Puthoff ML, Saskowski D. Reliability and responsiveness of gait speed, five times sit to stand, and hand grip strength for patients in cardiac rehabilitation. Cardiopulm Phys Ther J. 2013; 24: 31-37. PMID: 23754937
50. Mathiowetz V. Comparison of Rolyan and Jamar dynamometers for measuring grip strength. Occup. Ther. Int. 2002; 9(3): 201-9. doi: 10.1002/oti.165. PMID: 12374997
51. McCaffrey N, Kaambwa B, Currow DC, Ratcliffe J. Health-related quality of life measured using the EQ-5D-5L: South Australian population norms. Health Qual Life Outcomes. 2016; 14(1): 133. doi: 10.1186/s12955-016-0537-0. PMID: 27644755
52. Dams J, Rimane E, Steil R, et al. Reliability, validity and responsiveness of the EQ-5D-5L in assessing and valuing health status in adolescents and young adults with posttraumatic stress disorder: a randomized controlled trail. Psychiatr Q. 2021; 92(2): 459-471. doi: 10.1007/s11126-020-09814-6. PMID: 32803473
53. Rankin SL, Briffa TG, Morton AR, Hung J. A specific activity questionnaire to measure the functional capacity of cardiac patients. Am J Cardiol. 1996; 77(14): 1220-3. doi: 10.1016/s0002-9149(97)89157-6. PMID: 8651099
54. Krupp LB, LaRocca NG, Muir-Nash J, Steinberg AD. The fatigue severity scale: application to patients with multiple sclerosis and systemic lupus erythematosus. Arch Neurol. 1989; 46: 1121-1123. PMID: 2803071
55. Amtmann D, Bamer AM, Noonan V, et al. Comparison of the psychometric properties of two fatigue scales in multiple sclerosis. Rehabil Psychol. 2012; 57: 159-166. doi: 10.1037/a0027890. PMID: 22686554
56. de Morton NA, Davidson M, Keating JL. Validity, responsiveness and the minimal

clinically important difference for the de Morton mobility index (DEMMI) in an older acute medical population. BMC Geriatr. 2010; 10: 72. doi: 10.1186/1471-2318-10-72. PMID: 20920285

57. Wang J, Tan S, Gianotti L, Wu G. Evaluation and management of body composition changes in cancer patients. Nutrition. 2023;114:112132. doi:10.1016/j.nut.2023.112132.
58. Lee YS, Hong N, Witanto JN, Choi YR, Park J, Decazes P, et al. Deep neural network for automatic volumetric segmentation of whole-body CT images for body composition assessment. Clin Nutr. 2021;40(8):5038–5046. doi:10.1016/j.clnu.2021.06.025
59. Hurt RT, Ebbert JO, Croghan I, Nanda S, Schroeder DR, Teigen LM, et al. The comparison of segmental multifrequency bioelectrical impedance analysis and dual-energy X-ray absorptiometry for estimating fat free mass and percentage body fat in an ambulatory population. JPEN J Parenter Enteral Nutr. 2020;00(0):1–8. doi:10.1002/jpen.1994
60. Md Ali NA, El-Ansary D, Abdul Rahman MR, et al. Early Supervised Incremental Resistance Training (*ESpIRiT*) following cardiac surgery via a median sternotomy: a study protocol of a multicentre randomised controlled trial. BMJ Open. 2023; 13(7): e067914. doi: 10.1136/bmjopen-2022-067914. PMID: 37451709
61. Sterne JA, White IR, Carlin JB, Spratt M, Royston P, Kenward MG, et al. Multiple imputation for missing data in epidemiological and clinical research: potential and pitfalls. BMJ. 2009; 338:b2393. PMID:19564179
62. Serdar CC, Cihan M, Yucel D, Serdar MA. Sample size, power and effect size revisited: simplified and practical approaches in pre-clinical, clinical and laboratory studies.

Biochem Med (Zagreb). 2021; 31(1):010502. doi: 10.11613/BM.2021.010502 PMID:33380887

63. Gillen JB, Gibala MJ. Is high-intensity interval training a time-efficient exercise strategy to improve health and fitness? Appl Physiol Nutr Metab. 2014; 39: 409-412. doi:10.1139/apnm-2013-0187. PMID: 24552392

Appendix

Name/RN/Code: **Date:**

Patient's Personal Detail and Demographic

**Name/gender/age**	
**Address**	
**Contact number**	
**Occupation**	
**Level of education**	**Secondary school**<br><br>**University/college**
**Physical activities**	**Sedentary lifestyle**<br><br>**Active**<br><br>**Very active**
**Smoking Habit**	**Yes**<br><br>**No**<br><br>**Ex-smoker: since when**<br><br>**How many cigarette/day**

<table>
<tr><td>Underlying Medical

Illness</td><td>DM

HPT

Asthma

COPD

Cancer

CAD/IHD

Others</td></tr>
<tr><td>Past Surgical History</td><td></td></tr>
<tr><td>ASA</td><td><table>
<tr><th>ASA class</th><th>Physical status</th></tr>
<tr><td>1</td><td>Normal healthy patient</td></tr>
<tr><td>2</td><td>Patient with mild systemic disease</td></tr>
<tr><td>3</td><td>Patient with severe systemic disease</td></tr>
<tr><td>4</td><td>Patient with severe systemic disease that is a constant threat to life</td></tr>
<tr><td>5</td><td>Moribund patient not expected to survive without emergent procedure</td></tr>
</table></td></tr>
</table>

Bioimpedence Body Composition

Parameter	Score/measurement	Normal Range
**Weight (kg)**		
**Height (cm)**		
**BMI (kg/m2)**		
**Muscles mass (kg)**		
**Fat Mass (kg)**		
**Muscles Control (kg)**		
**Fitness Score**		
**Osseous**		
**Water**		

Inspiratory Muscles Assessment

	1st	2nd	3rd	Highest
MIP (cm2H2O)				
PIF (L/s)				

**Clinical Frailty Score : Circle only 1 answer**

Very Fit	Well	Managing well	Vulnerable	Mildly frail
Moderately frail	Severely frail	Very severely frail	Terminally ill	

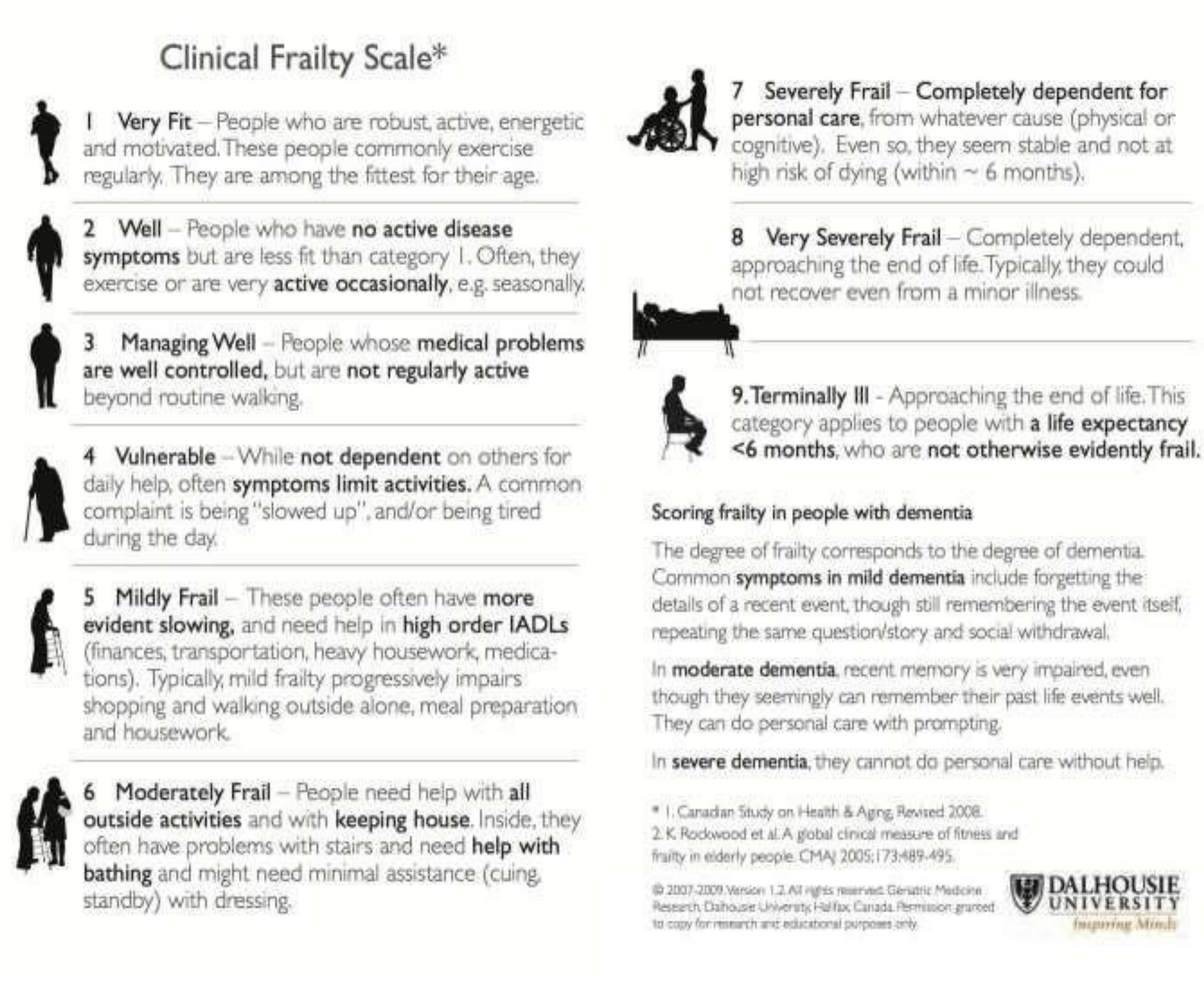

Clinical Frailty Scale*

1 **Very Fit** – People who are robust, active, energetic and motivated. These people commonly exercise regularly. They are among the fittest for their age.

2 **Well** – People who have **no active disease symptoms** but are less fit than category 1. Often, they exercise or are very **active occasionally**, e.g. seasonally.

3 **Managing Well** – People whose **medical problems are well controlled**, but are **not regularly active** beyond routine walking.

4 **Vulnerable** – While **not dependent** on others for daily help, often **symptoms limit activities.** A common complaint is being "slowed up", and/or being tired during the day.

5 **Mildly Frail** – These people often have **more evident slowing,** and need help in **high order IADLs** (finances, transportation, heavy housework, medications). Typically, mild frailty progressively impairs shopping and walking outside alone, meal preparation and housework.

6 **Moderately Frail** – People need help with **all outside activities** and with **keeping house**. Inside, they often have problems with stairs and need **help with bathing** and might need minimal assistance (cuing, standby) with dressing.

7 **Severely Frail** – **Completely dependent for personal care**, from whatever cause (physical or cognitive). Even so, they seem stable and not at high risk of dying (within ~ 6 months).

8 **Very Severely Frail** – Completely dependent, approaching the end of life. Typically, they could not recover even from a minor illness.

9. Terminally Ill - Approaching the end of life. This category applies to people with **a life expectancy <6 months**, who are **not otherwise evidently frail.**

Scoring frailty in people with dementia

The degree of frailty corresponds to the degree of dementia. Common **symptoms in mild dementia** include forgetting the details of a recent event, though still remembering the event itself, repeating the same question/story and social withdrawal.

In **moderate dementia**, recent memory is very impaired, even though they seemingly can remember their past life events well. They can do personal care with prompting.

In **severe dementia**, they cannot do personal care without help.

* 1. Canadian Study on Health & Aging, Revised 2008.
2. K. Rockwood et al. A global clinical measure of fitness and frailty in elderly people. CMAJ 2005;173:489-495.

© 2007-2009. Version 1.2. All rights reserved. Geriatric Medicine Research, Dalhousie University, Halifax, Canada. Permission granted to copy for research and educational purposes only.

DALHOUSIE UNIVERSITY
Inspiring Minds

Six Minute Walk Test Recording Sheet

Name:
Age:

Age Predicted Heart Rate maximum
210- (0.65xAGE):

Clinical note for using the recording sheet:
You do not need to record SpO_2%, heart rate and dyspnoea every minute but there is space on the table above if you wish to.

Walk 1
Date:
Time:

Walk 2
Date:
Time:

Walk 1				
	**BP**	**HR**	**SpO_2**	
PRE				
POST				
Comment				

Walk 2				
	**BP**	**HR**	**SpO_2**	
PRE				
POST				
Comment				

Time mins	SpO_2	HR	Dyspnoea	Rests
Rest				
1				
2				
3				
4				
5				
6				

Time mins	SpO_2	HR	Dyspnoea	Rests
Rest				
1				
2				
3				
4				
5				
6				

Recove ry 1				
2				

Recove ry 1				
2				

Distance 1:__________ Distance 2:___________

Limiting factor to the test:
SOB ☐ Low SpO_2 ☐
Legs fatigue ☐ other: _______

Limiting factor to the test:
SOB ☐ Low SpO_2 ☐
Legs fatigue ☐ other: _______

Hand Grip Strength Dominant Hand (Kg)

Please circle prior to test

RIGHT (1)

1st attempt	2nd attempt	3rd attempt	Best of three

LEFT (0)

1st attempt	2nd attempt	3rd attempt	Best of three

	Repetition
1 Minute STS	
30 mins STS	

Study ID ____________ Date ____________ Tester Initials ____________

SCORING:

A. Side-by-side-stand

Held for 10 sec ❒ 1 point

Not held for 10 sec ❒ 0 points

Not attempted ❒ 0 points

If 0 points, end Balance Tests

Number of seconds held if less than 10 sec: __.__ __sec

If participant did not attempt test or failed, circle why:

Tried but unable	1
Participant could not hold position unassisted	2
Not attempted, you felt unsafe	3
Not attempted, participant felt unsafe	4
Participant unable to understand instructions	5
Other (specify) ____________	6
Participant refused	7

B. Semi-Tandem Stand

Held for 10 sec ❒ 1 point

Not held for 10 sec ❒ 0 points

Not attempted ❒ 0 points *(circle reason above)*

If 0 points, end Balance Tests

Number of seconds held if less than 10 sec: __.__ __sec

C. Tandem Stand

Held for 10 sec ❒ 2 points

Held for 3 to 9.99 sec ❒ 1 point

Held for < than 3 sec ❒ 0 points

Not attempted ❒ 0 points (circle reason above)

Number of seconds held if less than 10 sec: __.__ __sec

D. Total Balance Tests score________(sum points)

Comments: ____

Study ID ________________ Date ______________ Tester Initials ______________

GAIT SPEED TEST SCORING:

Length of walk test course: Four meters ❒ Three meters ❒

A. Time for First Gait Speed Test (sec)

1. Time for 3 or 4 meters __ __.__ __sec
2. If participant did not attempt test or failed, circle why:
   Tried but unable 1
   Participant could not walk unassisted 2
   Not attempted, you felt unsafe 3
   Not attempted, participant felt unsafe 4
   Participant unable to understand instructions 5
   Other (Specify) ________________ 6
   Participant refused 7
   Complete score sheet and go to chair stand test

3. Aids for first walk...............None ❒ Cane ❒ Other ❒

Comments:____

B. Time for Second Gait Speed Test (sec)

1. Time for 3 or 4 meters __ __.__ __ sec
2. If participant did not attempt test or failed, circle why:
   Tried but unable 1
   Participant could not walk unassisted 2
   Not attempted, you felt unsafe 3
   Not attempted, participant felt unsafe 4
   Participant unable to understand instructions 5
   Other (Specify) 6
   Participant refused 7

3. Aids for second walk............ None ❒ Cane ❒ Other ❒

What is the time for the faster of the two walks?
Record the shorter of the two times __ __.__ __ sec
[If only 1 walk done, record that time] __ __.__ __ sec

If the participant was unable to do the walk: ❒ **0 points**

For 4-Meter Walk:		For 3-Meter Walk:	
If time is more than 8.70 sec:	❒ **1 point**	If time is more than 6.52 sec:	❒ **1 point**
If time is 6.21 to 8.70 sec:	❒ **2 points**	If time is 4.66 to 6.52 sec:	❒ **2 points**
If time is 4.82 to 6.20 sec:	❒ **3 points**	If time is 3.62 to 4.65 sec:	❒ **3 points**
If time is less than 4.82 sec:	❒ **4 points**	If time is less than 3.62 sec:	❒ **4 points**

Study ID ____________ Date ____________ Tester Initials ____________

SCORING

Single Chair Stand Test

	YES	NO
A. Safe to stand without help	❒	❒

B. Results:

Participant stood without using arms	❒	→ Go to Repeated Chair Stand Test
Participant used arms to stand	❒	→ End test; score as 0 points
Test not completed	❒	→ End test; score as 0 points

C. If participant did not attempt test or failed, circle why:

Tried but unable	1
Participant could not stand unassisted	2
Not attempted, you felt unsafe	3
Not attempted, participant felt unsafe	4
Participant unable to understand instructions	5
Other (Specify) ____________	6
Participant refused	7

Repeated Chair Stand Test

	YES	NO
A. Safe to stand five times	❒	❒

B. If five stands done successfully, record time in seconds.

Time to complete five stands __ __.__ __ sec

C. If participant did not attempt test or failed, circle why:

Tried but unable	1
Participant could not stand unassisted	2
Not attempted, you felt unsafe	3
Not attempted, participant felt unsafe	4
Participant unable to understand instructions	5
Other (Specify)	6
Participant refused	7

Scoring the Repeated Chair Test

Participant unable to complete 5 chair stands or completes stands in >60 sec:	❒ 0 points
If chair stand time is 16.70 sec or more:	❒ 1 points
If chair stand time is 13.70 to 16.69 sec:	❒ 2 points
If chair stand time is 11.20 to 13.69 sec:	❒ 3 points
If chair stand time is 11.19 sec or less:	❒ 4 points

Fatigue Severity Scale

Date: __ / __ / __ Patient Initial: Site Number

Please circle the number between 1 and 7 which you feel best fits the following statements. This refers to your usual way of life within the last week. 1 indicates "strongly disagree" and 7 indicates "strongly agree."

Read and circle a number	Strongly disagreed			Neither agreed nor Disagreed			strongly agree
1. My motivation is lower when I am fatigued	1	2	3	4	5	6	7
2. Exercise brings on my fatigue.	1	2	3	4	5	6	7
3. I am easily fatigued	1	2	3	4	5	6	7
4. Fatigue interferes with my physical functioning	1	2	3	4	5	6	7
5. Fatigue causes frequent problems for me	1	2	3	4	5	6	7
6. My fatigue prevents sustained physical functioning	1	2	3	4	5	6	7
7. Fatigue interferes with carrying out certain duties and responsibilities	1	2	3	4	5	6	7
8. Fatigue is among my most disabling symptoms.	1	2	3	4	5	6	7
9. Fatigue interferes with my work, family, or social life.	1	2	3	4	5	6	7
Total score<br>9-35 :Normal<br>> 35 : Exreme high level of fatique							

Patient Identified Cardiac Pain Using Numeric And Visual Prompts

Appendix C

Intensity of Chief Pain/Discomfort

How 'bad/distressing' is your pain/discomfort

Circle ONLY one number

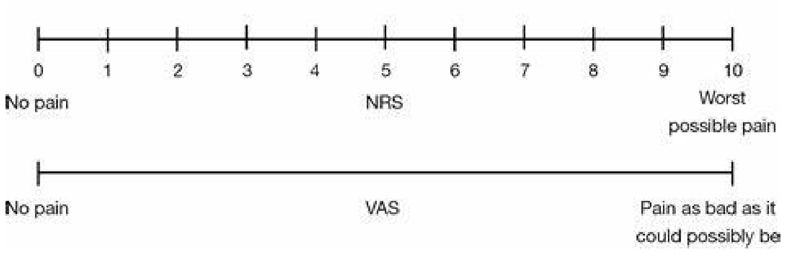

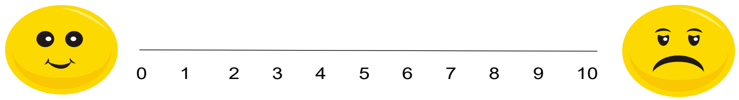

Global Rating of Change Scale

How does your function now, compare to your function at the initial assessment before surgery?

Instructions: Please Circle ONLY One number best describe your function past week

Global Rating of Change Scale
**1. Very much improved**<br>**2. Much improved**<br>**3. Minimally improved**<br>**4. No change**<br>**5. Minimally worse**<br>**6. Much worse**<br>**7. Very much worse**

SELF-ADMINISTERED PHYSICAL ACTIVITY QUESTIONNAIRE (SAQ)

Can you complete the following activities at a normal rate?

Circle YES or NO

		*
**a. Walk down a flight of stairs unassisted and without stopping?**	**YES**	**NO**
**b. Carry an 8kg weight (e.g. a load of wet washing) up 8 steps?**	**YES**	**NO**
**c. Do moderate gardening like weed or rake the leaves?**	**YES**	**NO**
**d. Walk briskly around an oval?**	**YES**	**NO**
**e. Carry at least 10kg (e.g. a suitcase) up 8 steps?**	**YES**	**NO**
**f. Carry objects that weigh at least 35kg (e.g. an 11 year old child)?**	**YES**	**NO**
**g. Do outdoor work like split wood or dig in the garden?**	**YES**	**NO**
**h. Participate in moderate activities like walk at a normal pace (4km/hr) or play golf and carry the clubs?**	**YES**	**NO**
**i. Participate in vigorous activities like swimming (crawl), jogging (8km/hr), cycling (17km/hr) or singles tennis?**	**YES**	**NO**

j. Do moderate work around the house like vacuum, sweep floors, or carry groceries? **YES** **NO**

k. Do heavy work around the house like strip and make the bed, hang out washing, or wash the car? **YES** **NO**

l. Push an electric or petrol mower on level ground? **YES** **NO**

m. Dress without stopping because of symptoms?

YES **NO**

*** If you answered "NO" to any of the above questions, what stops you doing these activities? (e.g. shortness of breath, angina, recent surgery etc.)**

Can you complete the following without symptoms?	*MET value*
**m.** Dress without stopping because of symptoms?	2.00
**j.** Do moderate work around the house like vacuum, sweep floors, or carry groceries?	2.50
**a.** Walk down a flight of stairs unassisted and without stopping?	3.00
**k.** Do heavy work around the house like strip and make the bed, hang out washing, or wash the car?	3.25
**c.** Do moderate gardening like weed or rake the leaves?	4.25
**l.** Push an electric or petrol mower on level ground?	4.50
**h.** Participate in moderate activities like walk at a normal pace (4km/hr) or play golf and carry the clubs?	4.75
**d.** Walk briskly around an oval?	5.00
**g.** Do outdoor work like split wood or dig in the garden?	5.50
**b.** Carry an 8kg weight (e.g. a load of wet washing) up 8 steps?	6.00
**e.** Carry at least 10kg (e.g. a suitcase) up 8 steps?	7.00

**f.**	Carry objects that weigh at least 35kg (e.g. an 11 year old child)?	7.50
**i.**	Participate in vigorous activities like swimming (crawl), jogging (8km/hr), cycling (17km/hr) or singles tennis?	9.00

MET = metabolic equivalent

Step 3: Record the patient's age in years, height in centimetres and weight in kilograms.

Step 4: To calculate VO_2; substitute these values into the following equation:

$$VO_2 = 2.36(SAQ) + 0.35(HEIGHT\ cm) - 0.19(AGE\ yrs) - 0.16(WEIGHT\ kg) - 33.89$$

Hospital Anxiety and Depression Scale (HADS)

Date: __ / __ / __ **Patient Initial:**

**Hospital Anxiety and Depression Scale**
Patients are asked to choose one response from the four given for each interview. They should give an immediate response and be dissuaded from thinking too long about their answers. The questions relating to anxiety are marked "A", and to depression "D". The score for each answer is given in the right column. Instruct the patient to answer how it currently describes their feelings.

A	**I feel tense or 'wound up':**	
	Most of the time	3
	A lot of the time	2
	From time to time, occasionally Not at	1
	all	0
D	**I still enjoy the things I used to enjoy:**	
	Definitely as much	0
	Not quite so much	1
	Only a little	2
	Hardly at all	3
A	**I get a sort of frightened feeling as if something awful is about to happen:**	
	Very definitely and quite badly	3
	Yes, but not too badly	2
	A little, but it doesn't worry me Not at	1
	all	0
D	**I can laugh and see the funny side of things:**	
	As much as I always could	0

	Not quite so much now	1
	Definitely not so much now	2
	Not at all	3
A	**Worrying thoughts go through my mind:**	
	A great deal of the time	3
	A lot of the time	2
	From time to time, but not too often	1
	Only occasionally	0
D	**I feel cheerful:**	
	Not at all	3
	Not often	2
	Sometimes	1
	Most of the time	0

A	**I can sit at ease and feel relaxed:**	
	Definitely	0
	Usually	1
	Not Often	2
	Not at all	3
D	**I feel as if I am slowed down:**	
	Nearly all the time	3
	Very often	2
	Sometimes	1
	Not at all	0
A	**I get a sort of frightened feeling like 'butterflies' in the stomach:**	

	Not at all	0
	Occasionally	1
	Quite Often	2
	Very Often	3
D	**I have lost interest in my appearance:**	
	Definitely	3
	I don't take as much care as I should	2
	I may not take quite as much care	1
	I take just as much care as ever	0
A	**I feel restless as I have to be on the move:**	
	Very much indeed	3
	Quite a lot	2
	Not very much	1
	Not at all	0
D	**I look forward with enjoyment to things:**	
	As much as I ever did	0
	Rather less than I used to	1
	Definitely less than I used to	2
	Hardly at all	3
A	**I get sudden feelings of panic:**	
	Very often indeed	3
	Quite often	2
	Not very often	1
	Not at all	0
D	**I can enjoy a good book or radio or TV program:**	
	Often	0
	Sometimes	1

	Not often	2
	Very seldom	3

Total Anxiety (A) score:

Total Depression (D) score:

Total Score (A+D

EQ-5D-5L

Health Questionnaire

English version for Malaysia

Under each heading, please tick the ONE box that best describes your health TODAY.

MOBILITY

I have no problems in walking about ❑

I have slight problems in walking about ❑

I have moderate problems in walking about ❑

I have severe problems in walking about ❑

I am unable to walk about ❑

SELF-CARE

I have no problems cleaning my body or dressing myself ❑

I have slight problems cleaning my body or dressing myself ❑

I have moderate problems cleaning my body or dressing myself ❑

I have severe problems cleaning my body or dressing myself ❑

I am unable to clean my body or dress myself ❑

USUAL ACTIVITIES *(e.g. work, study, housework, family or leisure activities)*

I have no problems doing my usual activities ❑

I have slight problems doing my usual activities ❑

I have moderate problems doing my usual activities ❑

I have severe problems doing my usual activities ❑

I am unable to do my usual activities ❑

PAIN / DISCOMFORT

I have no pain or discomfort ❑

I have slight pain or discomfort ❑

I have moderate pain or discomfort ❑

I have severe pain or discomfort ❑

I have extreme pain or discomfort ❑

ANXIETY / DEPRESSION

I am not anxious or depressed ❑

I am slightly anxious or depressed ❑

I am moderately anxious or depressed ❑

I am severely anxious or depressed ❑

I am extremely anxious or depressed ❑

We would like to know how good or bad your health is TODAY.

The best health you can

This scale is numbered from 0 to 100.

100 means the best health you can imagine.
0 means the worst health you can imagine.

Mark an X on the scale to indicate how your health is TODAY.

Now, please write the number you marked on the scale in the box below.

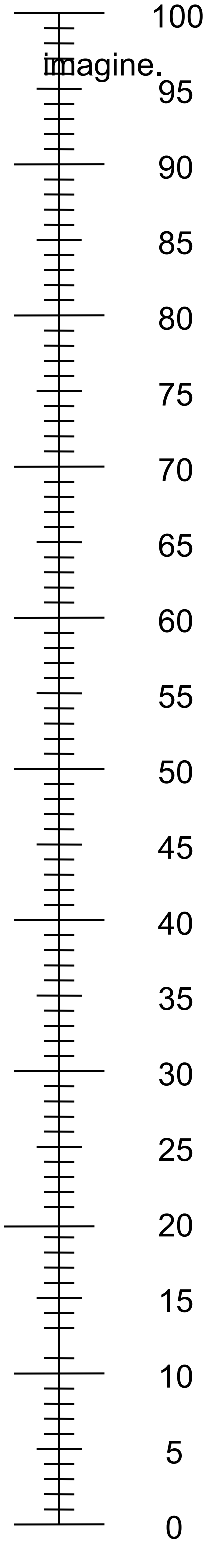

YOUR HEALTH TODAY = ☐

The worst health you can imagine

UNIVERSITI KEBANGSAAN MALAYSIA
The National University of Malaysia

SEKRETARIAT ETIKA PENYELIDIKAN UKM • UKM RESEARCH ETHICS SECRETARIAT

Reference: UKM PPI/111/8
Date : 12 November 2024

Associate Professor Dato' Dr. Nur Ayub Md Ali
Department of Surgery
Faculty of Medicine UKM

Dear Professor/Datuk/Dato'/Datin/Sir/Madam,

ETHICAL APPROVAL TO CONDUCT RESEARCH IN THE NATIONAL UNIVERSITY OF MALAYSIA

Research title	:	The Effectiveness Of High-Intensity Interval Training Versus Moderate Intensity Continous Training In Prehabilitation Among Patients Undergoing Upper Abdominal Surgery
Ethics Ref. No.	:	JEP-2024-527
Approval period	:	06 November 2024 – 05 November 2026
Study Site	:	Hospital Canselor Tuanku Muhriz
Sample Size	:	74 for Web Survey<br>60 for RCT

With reference to the above.

2. The Research Ethics Committee, The National University of Malaysia (RECUKM) has provided ethical approval for above study. Please be reminded permission from the Deputy Dean of research of the faculty or Director of Institute/Centre and all relevant heads of departments / units where the study will be carried out must be obtained prior to the study. You are required to follow and comply with their decision and all other relevant regulations.

3. Please note that the investigator's responsibility is to ensure that:

i. All Adverse Events should be reported to RECUKM as soon as possible.
ii. Progress report should be submitted every **6 Months.**
iii. All changes / amendments to the study documents / study sites / study team must be notified to the RECUKM. All changes must be approved by RECUKM before continuation of the study.
iv. Application for renewal of the approval has to be submitted to RECUKM within one month (1 month) prior to the expiry of ethical approval.
v. Final Report should be provided to RECUKM when the project is complete.
vi. Please take note that all records and data are to be kept strictly CONFIDENTIAL and can only be used for the purpose of this study. All precautions are to be taken to maintain data confidentiality.

Required forms can be obtained from the The Research Ethics Committee, The National University of Malaysia (RECUKM) website: **https://www.ukm.my/jepukm**

Thank you.

Sekretariat Etika Penyelidikan Universiti Kebangsaan Malaysia
Tingkat 1, Blok Klinikal, Hospital Canselor Tuanku Muhriz,Pusat Perubatan UKM, Jalan Yaacob Latif, Bandar Tun Razak, 56000 Cheras Kuala Lumpur.
Telefon: +603-9145 5046 / 5048
Email: sepukm@ukm.edu.my Web: https://www.ukm.my/jepukm/

Yours sincerely,

PROFESOR DR MOHD SHAHRIR MOHAMED SAID
Chairman
Research Ethics Committee
The National University Of Malaysia

c.c.
- **Director**
  Hospital Canselor Tuanku Muhriz

- **Deputy Dean (Research & Innovation)**
  Faculty of Medicine, UKM

- **Head of Department Surgery**
  Faculty of Medicine UKM

- **Dr. Nor Azura Azmi**
  Center for Rehabilitation and Special Needs Studies (ICaRehab)
  Faculty of Health Sciences

- **Dr. Katijjahbe Mohd Ali**
  Medical Rehabilitation Services
  Hospital Canselor Tuanku Muhriz
  UKM Medical Centre

- **Dr. Chik Ian**
- **Dr. Suriah Ahmad (P138488 – Master Candidate)**
  Department of Surgery
  Faculty of Medicine UKM

- **Dr. Syarifah Noor Nazihah Syed Masri**
  Department of Anaesthesiology & Intensive Care
  Hospital Canselor Tuanku Muhriz
  UKM Medical Centre

- Approval letter File 2024

UNIVERSITI KEBANGSAAN MALAYSIA
The National University of Malaysia

SEKRETARIAT ETIKA PENYELIDIKAN UKM • UKM RESEARCH ETHICS SECRETARIAT

**NAME OF ETHICS COMMITTEE/IRB:**<br>Research Ethics Committee,<br>The National University of Malaysia	**ETHICS COMMITTEE/IRB REF NO : JEP-2024-527**
**PROTOCOL TITLE:**<br>The Effectiveness of High-Intensity Interval Training versus Moderate Intensity Continous Training in Prehabilitation among Patients Undergoing Upper Abdominal Surgery.	
**PRINCIPAL INVESTIGATOR:**<br>Associate Professor Dato' Dr. Nur Ayub Md Ali<br>Department of Surgery<br>Faculty of Medicine UKM	

The following items [X] have been received and reviewed in connection with the above study to be conducted by the above investigator.

Documents

[X] Research Application Form (UKM-JEP-BO01)
[X] Review Form (UKM-JEP-BO02)
[X] Non-Disclosure Agreement (UKM-JEP-BO03)
[X] Conflict Of Interest Form
[X] Research Proposal / Protocol
[X] Participant Information Sheet:
    [X] Malay [X] English [ ] Others :
[X] Participant Consent Form:
    [X] Malay [X] English [ ] Others :
[ ] Questionnaire:
    [ ] Malay [ ] English [ ] Others :
[X] Curriculum Vitae of Researchers:
    [X] Principal [X] Co-researcher [X] Student
[X] Good Clinical Practice Certificate (GCP)
[ ] Project Agreement

The Research Ethics Committee, The National University of Malaysia operates in accordance to the International Conference of Harmonization Good Clinical Practice Guidelines.

Comments (if any): ______________________________

Date of Approval: 06 November 2024

PROFESSOR DR. MOHD SHAHRIR MOHAMED SAID
Chairman
Research Ethics Committee
The National University of Malaysia

Sekretariat Etika Penyelidikan Universiti Kebangsaan Malaysia
Tingkat 1, Blok Klinikal, Hospital Canselor Tuanku Muhriz,Pusat Perubatan UKM, Jalan Yaacob Latif, Bandar Tun Razak, 56000 Cheras Kuala Lumpur.
Telefon: +603-9145 5046 / 5048
Email: sepukm@ukm.edu.my Web: https://www.ukm.my/jepukm/

ILMU, MUTU DAN BUDI

UNIVERSITI KEBANGSAAN MALAYSIA
The National University of Malaysia

SEKRETARIAT ETIKA PENYELIDIKAN UKM • *UKM RESEARCH ETHICS SECRETARIAT*

Ref: UKM PPI/111/8
Date: 24 **June 2025**

Associate Professor Dato' Dr. Nur Ayub Md Ali
Department of Surgery
Faculty of Medicine UKM

ETHICAL APPROVAL OF PROPOSAL AMENDMENT

Research Title : The Effectiveness of High-Intensity Interval Training versus Moderate Intensity Continous Training in Prehabilitation among Patients Undergoing Major Abdominal Surgery

Ethics Ref. No. : JEP-2024-527

Approval period : 06 November 2024 – 05 November 2026

The above matter mentioned and your letter dated 18 April 2025 is referred.

2. The Research Ethics Committee, The National University of Malaysia has approved your proposal amendment as title above in the Research Ethics Committee Meeting No. 11/2025 dated 12 June 2025 as below:

No	Old Ttile	New Ttile
i	The Effectiveness of High-Intensity Interval Training versus Moderate Intensity Continous Training in Prehabilitation among Patients Undergoing Upper Abdominal Surgery.	The Effectiveness of High-Intensity Interval Training versus Moderate Intensity Continous Training in Prehabilitation among Patients Undergoing Major Abdominal Surgery" (page 1)
Ii	Data collection (page 26)	

3. Please submit any **Adverse Events Report**, **Progress Report every 6 months** and **Final Report** upon completion of the research to the Research Ethics Committee. The minute of meeting no. 11/2025 dated 12 June 2025 is attached for your reference.

Thank you.

Yours sincerely,

PROFESOR DR MOHD SHAHRIR MOHAMED SAID
Chairman
Research Ethics Committee
The National University of Malaysia

c.c. - **Director**
Hospital Canselor Tuanku Muhriz

- **Deputy Dean (Research & Innovation)**
Faculty of Medicine, UKM

- **Head of Department Surgery**
Faculty of Medicine UKM

- **Dr. Nor Azura Azmi**
Center for Rehabilitation and Special Needs Studies (ICaRehab)
Faculty of Health Sciences

- **Dr. Katijjahbe Mohd Ali**
Medical Rehabilitation Services
Hospital Canselor Tuanku Muhriz
UKM Medical Centre

- **Dr. Chik Ian**
- **Dr. Suriah Ahmad (P138488 – Master Candidate)**
Department of Surgery
Faculty of Medicine UKM

- **Dr. Syarifah Noor Nazihah Syed Masri**
Department of Anaesthesiology & Intensive Care
Hospital Canselor Tuanku Muhriz
UKM Medical Centre

- Approval letter File 2024

UNIVERSITI KEBANGSAAN MALAYSIA

The National University of Malaysia

MINUTES OF MEETING
RESEARCH ETHICS COMMITTEE
THE NATIONAL UNIVERSITY OF MALAYSIA
NO.11/2025

Date	:	12 June 2025 (Thursday)
Time	:	2.15 p.m
Application	:	Zoom Cloud Meeting

8. **Presentation list changes / Amendment after approval**

8.1 Endorsement for Protocol Amendment after approval from 22 May 2025 – 11 June 2025.

8.1.1 The protocol amendment lists were approved.

Ref: UKM PPI/111/8
Date: 06 **August 2025**

Associate Professor Dato' Dr. Nur Ayub Md Ali
Department of Surgery
Faculty of Medicine UKM

ETHICAL APPROVAL OF PROPOSAL AMENDMENT

Research Title : The Effectiveness of High-Intensity Interval Training versus Moderate Intensity Continous Training in Prehabilitation among Patients Undergoing Major Abdominal Surgery

Ethics Ref. No. : JEP-2024-527

Approval period : 06 November 2024 – 05 November 2026

The above matter mentioned and your letter dated 07 July 2025 is referred.

2. The Research Ethics Committee, The National University of Malaysia has approved your proposal amendment as title above in the Research Ethics Committee Meeting No. 15/2025 dated 24 July 2025 as below:

i. The sample size has been revised from 60 to 70 participants following a recalculation (page 25).
ii. The inclusion criterion requiring participants to achieve a six-minute walk distance (6MWD) of at least 300 meters has been removed. This change is due to the limited number of eligible patients and the absence of this criterion in previous related studies (page 31).
iii. The age range for participants has been updated from 18–75 years to 18 years and above to allow for a wider recruitment pool (page 24).
iv. The screening for the Hospital Anxiety and Depression Scale (HADS) has been removed, as HADS is included as one of the outcome measures in the study (Page 31)

3. Please submit any **Adverse Events Report**, **Progress Report every 6 months** and **Final Report** upon completion of the research to the Research Ethics Committee. The minute of meeting no. 15/2025 dated 24 July 2025 is attached for your reference.

Thank you.

Yours sincerely,

PROFESOR DR MOHD SHAHRIR MOHAMED SAID
Chairman
Research Ethics Committee
The National University of Malaysia

c.c. - **Director**
Hospital Canselor Tuanku Muhriz

- **Deputy Dean (Research & Innovation)**
  Faculty of Medicine, UKM

- **Head of Department Surgery**
  Faculty of Medicine UKM

- **Dr. Nor Azura Azmi**
  Center for Rehabilitation and Special Needs Studies (ICaRehab)
  Faculty of Health Sciences

- **Dr. Katijjahbe Mohd Ali**
  Medical Rehabilitation Services
  Hospital Canselor Tuanku Muhriz
  UKM Medical Centre

- **Dr. Chik Ian**
- **Dr. Suriah Ahmad (P138488 – Master Candidate)**
  Department of Surgery
  Faculty of Medicine UKM

- **Dr. Syarifah Noor Nazihah Syed Masri**
  Department of Anaesthesiology & Intensive Care
  Hospital Canselor Tuanku Muhriz
  UKM Medical Centre

- Approval letter File 2024

UNIVERSITI KEBANGSAAN MALAYSIA

The National University of Malaysia

MINUTES OF MEETING
RESEARCH ETHICS COMMITTEE
THE NATIONAL UNIVERSITY OF MALAYSIA
NO. 15/2025

Date	:	24 July 2025 (Thursday)
Time	:	2.15 p.m
Application	:	Zoom Cloud Meeting

8. Presentation list changes / Amendment after approval

8.1 Endorsement for Protocol Amendment after approval from 10 July 2025 – 23 July 2025.

8.6.1 The lists were approved.
