# Supplementary material for: The effectiveness of high-intensity interval training versus moderate intensity continuous training in prehabilitation among patients undergoing major abdominal surgery: A study protocol
Source: PLoS One. 2025 Oct 10;20(10):e0332361. doi: 10.1371/journal.pone.0332361 (PMC12513630; doi:10.1371/journal.pone.0332361)
Supplement: Attachment — Submitted filename: Rebuttal Letter Response to Reviewers.pdf [file pone.0332361.s005.pdf]

Editor-in-Chief
PLOS ONE 9th August 2025

Dear Editor-in-Chief,

I am pleased to resubmit our revised manuscript entitled ***"The effectiveness of high-intensity interval training versus moderate intensity continuous training in prehabilitation among patients undergoing major abdominal surgery: A study protocol"*** **(PONE-D-25-08010) for further consideration in PLOS ONE.**

We would like to thank you and the reviewers for the constructive feedback provided on our original submission. We have carefully addressed all comments and suggestions. We believe the revisions have significantly improved the clarity, scientific rigor, and presentation of the manuscript. A detailed, point-by-point response to each comment is provided in **the table response to the reviewers** below, along with marked and clean versions of the revised manuscript.

In addition to addressing the reviewers' comments, we would like to inform you of several amendments to the study protocol that were made prior to and independent of the reviewers' feedback, and which have been approved by our institutional ethics committee. Firstly, we have revised our study population from patients undergoing "upper abdominal surgery" to those undergoing "**major abdominal surgery**". This change was made primarily due to improved accessibility to an adequate sample size, which is crucial for ensuring the statistical power and generalizability of our findings. Additionally, the broader classification of major abdominal surgery still encompasses upper abdominal procedures and allows for a more inclusive representation of surgical patients who may benefit from prehabilitation. This adjustment aligns with similar protocols in existing literature and supports the feasibility of recruitment within the study timeframe. This amendment to the study population has been approved by the institutional ethics committee (please refer to the letter of ethical approval of the proposal amendment, 24th June 2025).

Secondly, we also revised the study's sample size from n= 60 to **n=70,** a decision made prior to receiving reviewer feedback. Additional amendments include:

i) **Removal of the inclusion criterion requiring participants to achieve a 6-minute walk distance of at least 300 m**, due to the limited number of eligible patients and the absence of this requirement in previous related studies;
ii) The age range for participants has been updated from 18-75 years to **18 years and above** to broaden the recruitment pool,

iii) **Removal of the Hospital Anxiety and Depression Scale (HADS) as a screening tool**, as HADS will be used as one of the outcome measures in this study.

These amendments have been reviewed and approved by the institutional ethics committee (please refer to the letter of ethical approval of the proposal amendment, 6th August 2025).

Below is the table that response to the reviewers.

i. Editor comment

No	Editor comment	Correction
1.	Please address the statistical plan	The statistical plan for data analysis has been revised. Please refer to page 19-20.

ii. Reviewer # 1

No.	Reviewer comments	Corrections
1.	I think your sample size calculation is currently wrong. We usually do not use type I error rate of 0.9 in a sample size calculation.	Thank you for your feedback. The sample size was recalculated with the type 1 error rate of 0.8 and alpha of 0.05. The standard deviation of 6MWT has been included.<br><br>Please refer to page 9.
2.	In addition to effect size of 30 meters difference in 6MWT, you also need to have a sensible value for the standard deviation. The results of only requiring 46 subjects probably are based on the assumption that you have very strong effect and very low variability.	Thank you for your insightful comment. We acknowledge the importance of justifying both the effect size and the standard deviation (SD) in our sample size calculation. Therefore, the recalculation of sample size using the recent systematic review of prehabilitation in patients waiting for major abdominal surgery (Alsuwaylihi et al. 2024) has been done.<br><br>The sample size was calculated based on the primary outcome measure (6MWT). A recent systematic review of prehabilitation in patients undergoing

		major abdominal surgery reported significant improvement in 6MWT, with a mean difference of 29.4 meters (95% CI: 5.6 to 53.3 m; p = 0.02) compared to standard care [31]. The estimated standard deviation was approximately 101.6 meters, corresponding to an effect size (Cohen's *d*) of 0.29. Using this effect size, with α of 0.05 and power of 80%, the required sample size was calculated to be 54 participants. Allowing for a 30% dropout rate, a total of 70 participants (35 per group) will be recruited. Sample size calculation was performed using G*Power version 3.1.9.7.<br><br>Please refer to page 9.
3.	The statistical analysis is a typical longitudinal data analysis plan and looks straightforward to me.	Thank you for your positive feedback regarding the statistical analysis plan. In response to your comment, we have revised the analysis approach from a two-way repeated measures ANOVA to a mixed model ANOVA to enhance robustness. This method allows for better handling of missing data, accommodates individual variability through random effects, and provides greater flexibility for longitudinal data analysis.<br><br>Please refer to page 19-20.
4.	Change "two-tailed" to "two-sided" on page 19	'Two-tailed' has been changed to 'two-sided' on page 20.
5.	Are there any potential side effects that you need to discuss?	Yes, the potential side effects have been discussed in page 22, as below:<br><br>Any potential adverse effects during exercise sessions, including musculoskeletal strain, cardiovascular events or respiratory distress will be closely monitored in both groups. Adverse events will be systematically

		recorded and reported. All exercise sessions will be conducted under the supervision of experienced physiotherapists, and participants will be screened for contraindications to exercise before each session to ensure safety.

iii. Reviewer # 2

No.	Reviewer comments	Corrections
1.	The max HR formula/ Karvonen method of calculation may not be suitable for all patient groups, there are other formulas which may be more suitable and considers past medical history such as if there are on beta blockers or not. This concerns safety, and is a necessary consideration	Thank you for your valuable feedback. We acknowledge that the Karvonen formula may not be suitable for all patient populations, particularly those on beta-blockers or with underlying cardiac conditions. In such cases, training intensity will be guided by the patient's rating of perceived exertion (RPE) which 10-13 RPE for MICT and 16-18 RPE for HIIT, and appropriate modifications will be made to ensure safety. This approach allows for individualized intensity adjustment while maintaining the safety and effectiveness of the intervention.<br><br>Please refer to page 11-12.
2.	Other outcome measures such as 10m walk test ie walking speed can be considered. And also 1 minute STS vs 30s STS vs FTSTS has different advantages. Unless the patients have COPD?	Thank you for your feedback. We considered the 1-minute sit-to-stand test in this protocol due to its superior sensitivity over the 30-second or FTSTS in assessing endurance-related functional capacity, especially in populations with comorbid of pulmonary complications, which is common in major abdominal surgery patients.<br><br>Please refer to page 14.

3.	Noted that PRE is a component of the exercise programme but there are no components of strength in the pre and post outcome measurement	Upper limb strength will be assessed using a hand-held dynamometer, following standardized protocols. Three trials will be conducted and the peak force (kg) will be recorded. Please refer to page 15.
4.	May I check: Where is this exercise programme adopted from?	The prehabilitation exercise program was adapted from previously published protocols used in preoperative populations [Boden 2024; Boden et al. 2018; Boden & Denehy 2022; Tew et al. 2019; Md Ali et al. 2023] with modifications tailored to the local clinical setting and characteristics of the target patient population. This prehabilitation program is designed based on recommendations from the current ERAS guidelines for major abdominal surgery.<br><br>Please refer to page 10.
5.	This exercise programme has multi components- including PRE and IMT, but the only difference between the the 2 groups are the aerobic component? Please clearly state under the intervention group, that they will also be receiving the same PRE and IMT training if so.	Thank you for your feedback.<br>The exercise programme has been rewrite clearly stated under the intervention group which include component of high intensity aerobic training and the same PRE and IMT training as standard care group.<br><br>Please refer to page 12.

We sincerely appreciate your time and consideration, and we look forward to the opportunity to contribute to the literature through publication in PLOS ONE.

Yours sincerely,

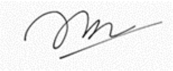

Nor Azura Azmi (PhD)
Physiotherapy Programme, Center for Rehabilitation and Special Needs Studies,
Faculty of Health Science, Universiti Kebangsaan Malaysia, Malaysia.
Email: nazura.azmi@ukm.edu.my
Phone: +6011-20971716
